# Thalidomide Inhibits Human iPSC Mesendoderm Differentiation by Modulating CRBN-dependent Degradation of SALL4

**DOI:** 10.1038/s41598-020-59542-x

**Published:** 2020-02-18

**Authors:** David G. Belair, Gang Lu, Laura E. Waller, Jason A. Gustin, Nathaniel D. Collins, Kyle L. Kolaja

**Affiliations:** 1 Nonclinical Development, Celgene Corporation, Summit, NJ USA; 2 Protein Homeostasis, Celgene Corporation, San Diego, CA USA; 3 Cell Design Studio, MilliporeSigma, St. Louis, MO USA

**Keywords:** Genetic engineering, High-throughput screening, Stem-cell differentiation, Toxicology

## Abstract

Exposure to thalidomide during a critical window of development results in limb defects in humans and non-human primates while mice and rats are refractory to these effects. Thalidomide-induced teratogenicity is dependent on its binding to cereblon (CRBN), the substrate receptor of the Cul4A-DDB1-CRBN-RBX1 E3 ubiquitin ligase complex. Thalidomide binding to CRBN elicits subsequent ubiquitination and proteasomal degradation of CRBN neosubstrates including SALL4, a transcription factor of which polymorphisms phenocopy thalidomide-induced limb defects in humans. Herein, thalidomide-induced degradation of SALL4 was examined in human induced pluripotent stem cells (hiPSCs) that were differentiated either to lateral plate mesoderm (LPM)-like cells, the developmental ontology of the limb bud, or definitive endoderm. Thalidomide and its immunomodulatory drug (IMiD) analogs, lenalidomide, and pomalidomide, dose-dependently inhibited hiPSC mesendoderm differentiation. Thalidomide- and IMiD-induced SALL4 degradation can be abrogated by CRBN V388I mutation or SALL4 G416A mutation in hiPSCs. Genetically modified hiPSCs expressing CRBN E377V/V388I mutant or SALL4 G416A mutant were insensitive to the inhibitory effects of thalidomide, lenalidomide, and pomalidomide on LPM differentiation while retaining sensitivity to another known limb teratogen, all-trans retinoic acid (atRA). Finally, disruption of LPM differentiation by atRA or thalidomide perturbed subsequent chondrogenic differentiation *in vitro*. The data here show that thalidomide, lenalidomide, and pomalidomide affect stem cell mesendoderm differentiation through CRBN-mediated degradation of SALL4 and highlight the utility of the LPM differentiation model for studying the teratogenicity of new CRBN modulating agents.

## Introduction

Exposure to thalidomide during a defined period of early fetal development results in a high incidence of phocomelia in humans. Several mechanisms for thalidomide-induced teratogenicity have been proposed^1^. Prior to 2010, the putative cellular and molecular teratogenic mechanism was that thalidomide inhibits angiogenesis^2,3^ by binding to GC-rich gene promoters^4^ and thereby blocking transcription of IGF-1 and FGF-2^5^. Inhibition of angiogenesis was hypothesized to disrupt limb outgrowth by impairing morphogen signaling critical to the developing limb. In 2010, Ito *et al*. demonstrated that thalidomide binds to cereblon (CRBN), a component of the E3 ubiquitin ligase complex with DDB1 and CUL4A, and inhibits autoubiquitination^6^, suggesting that thalidomide could induce teratogenicity by modifying CRBN-mediated degradation of substrates.

Thalidomide teratogenicity is species-dependent, as humans and non-human primates (NHPs) are sensitive to thalidomide-induced phocomelia whereas rodents are insensitive^1,7^. A crystal structure of thalidomide in complex with human and mouse CRBN provides a structural basis for the lack of sensitivity to thalidomide in rodents^8^. The thalidomide-binding domain on CRBN is highly conserved among sensitive species (*e.g*. NHPs and humans), whereas mice and rats harbor two mutations in the thalidomide-binding domain, E377V and V388I, that interfere with thalidomide-induced ubiquitination and subsequent CRBN-mediated degradation of substrates^9^. Thalidomide was recently shown to induce CRBN-dependent degradation of the transcription factor SALL4^10,11^. De-activating mutations in SALL4 produce thalidomide-like embryonic limb defects in humans^12^ and mice^13^, which suggests that thalidomide-induced degradation of SALL4 may be an essential component of thalidomide-induced teratogenicity. SALL4 possesses a conserved structural degron in its second C2H2 zinc finger that mediates thalidomide-induced CRBN binding, and G416A mutation in this region blocks thalidomide-induced SALL4 ubiquitination and degradation^10,11^. We investigated degradation of SALL4 as a putative mechanism of thalidomide-induced limb teratogenicity in a novel phenotypic *in vitro* assay of limb development.

Thalidomide has been hypothesized to induce phocomelia through disruption of cell and tissue morphogenesis processes during formation of the limb: inhibition of cell migration from the somatopleure to the limb bud^14^, inhibition of limb mesenchyme proliferation^15^, and inhibition of limb angiogenesis^3^. The discovery and characterization of human embryonic stem cells and human pluripotent stem cells has enabled interrogation of the cellular mechanisms of thalidomide-induced limb teratogenicity. The gold standard *in vitro* teratogenicity screening assay is the mouse embryonic stem cell test (mEST), which measures the viability and spontaneous cardiac differentiation of mouse embryonic stem cells (mESCs) cultured as embryoid bodies. Meta-analyses of non-, weak, and strong embryotoxicants have characterized the accuracy of the mEST as between 53–79% for predicting *in vivo* embryotoxicity^16,17^. However, the mEST is notably insensitive to the effects of thalidomide and only exhibits nonspecific toxicity to thalidomide exposure above 400 µM^18^, which is orders of magnitude higher than the Cmax of thalidomide in humans^7^. The insensitivity of the mEST to thalidomide is a concordant result as thalidomide exhibits species-specific teratogenicity and does not elicit limb malformations in mice.

Human pluripotent stem cells have proven useful in investigating the teratogenicity of thalidomide *in vitro*. Studies on the effect of thalidomide on human pluripotent stem cells (hPSCs) characterized cytotoxicity in undifferentiated hPSCs^19,20^ and hPSC-derived mesoderm^21^ and functional effects on hPSC-derived mesendoderm^22^ and cardiac mesoderm^20^ at concentrations above 100 µM. However, these studies demonstrated effects of thalidomide on PSCs or PSC-derived mesoderm *in vitro* at concentrations >5-fold in excess of the thalidomide clinical Cmax^7^, which raises concern regarding the relevance of these effects to thalidomide teratogenicity. A novel hPSC-based screening assay of definitive endoderm differentiation demonstrated 94% accuracy in predicting visceral malformations induced by a selection of compounds and was highly sensitive to the effect of thalidomide at non-cytotoxic concentrations below 1 µM^23^. Although the above studies show thalidomide may interfere with mesoderm specification or function, they do not elucidate a definitive molecular mechanism of the teratogenicity of thalidomide, lenalidomide, and pomalidomide. Here we examined the SALL4-dependence of stem cell differentiation in two distinct phenotypic assays of mesendoderm differentiation using hiPSCs.

During embryonic development, the limb bud originates from the lateral plate mesoderm (LPM), which subdivides into the somatic and splanchnic mesoderm, the former of which initiates the development of the limb bud^24^. LPM cells migrate to the limb fields and undergo condensation, proliferation, and ultimately chondrogenesis to cartilaginous elements that ossify to form the stylopod, zeugopod, and autopod skeletal elements of developing limbs^25^. Differentiation to a LPM-like cell phenotype was demonstrated with mouse^26^ and human pluripotent stem cells^27,28^. Further, mESC aggregates that were differentiated to LPM-like cells engraft and contribute to developing limbs^29^ and regenerating mouse phalanges^26^, highlighting the biological relevance of stem cell-derived LPM. Using a protocol adapted from literature describing LPM differentiation *via* GSK3 inhibition^27^, LPM differentiation of hiPSCs over the course of 2 days here was characterized by induction of FOXF1 and loss of NANOG by qPCR and high content imaging. The LPM differentiation assay was sensitive to treatment with thalidomide, lenalidomide, and pomalidomide. Interfering with CRBN-mediated SALL4 degradation through genetic engineering rendered hiPSCs insensitive to treatment with either thalidomide, lenalidomide, or pomalidomide in both a definitive endoderm differentiation assay and the LPM differentiation assay described here. Our results present evidence of a phenotypic link between thalidomide and IMiD-induced degradation of SALL4 and inhibition of key transcription factors involved in development of the mesendoderm.

## Materials and Methods

### Cell culture

Two commercially available hiPSC lines were used here. The female human episomal hiPSC line was purchased from Gibco, and the XCL-1 male hiPSC line from XCell Science was licensed for use *via* an agreement with MilliporeSigma. Gibco hiPSCs were used for development and characterization of the LPM differentiation assay, while the XCL-1 hiPSC line was used for the genetic engineering as described below and for characterization with both the LPM differentiation assay and the definitive endoderm (DE) differentiation assay. Gibco and XCL-1 hiPSC lines were cultured in mTeSR-1 (Stem Cell Technologies) on plates that were pre-coated with hESC-qualified Matrigel (Corning). Passaging the hiPSCs into small clumps was carried out by incubating hiPSCs with Versene (Gibco) for 4.5 min at 37 °C, 95% RH, and 5% CO_2_ (hereafter referred to as ‘normal culture conditions’). After dissociation, Versene was aspirated, and hiPSCs were resuspended in mTeSR-1. A small aliquot of hiPSCs was centrifuged at 200 × g for 5 min, resuspended in 0.25% trypsin (Gibco), and incubated for 5 min under normal culture conditions. The resulting single-cell suspension was then diluted 1:1 in hES-qualified FBS (Gibco), diluted 1:1 in Trypan blue, and counted on Countess II automated cell counter (Invitrogen). hiPSCs were seeded as small clumps as described below for each assay. Passaging the hiPSCs into a single-cell suspension was carried out as described previously for the SOX17 definitive endoderm differentiation assay^23^.

### Generation and characterization of hiPSCs

Genetically engineered hiPSC clones were generated and characterized by the Millipore-Sigma Cell Design Studio. Genetic engineering was carried out on the NCRM-1 NIH CRM control hiPSC line (male), commercially available as the XCL-1 line (XCell Science). Starting hiPSC cultures were of high quality and cultured to moderate density (70–80%) and were visually absent of spontaneously differentiated cells. For nucleofection, cells were in exponential log phase.

### SALL4 G416A genetic engineering

sgRNAs were designed to target the strong ubiquitination site of the SALL4 gene. XCL-1 hiPSCs were co-transfected with an ssODN repair template, together with Cas9/gRNA RNP (targeting genomic region **CCT**CAAGGTGCACTTTCACCGA with protospacer adjacent motif, PAM, (in bold font)) complex targeting SALL4 in human iPSC. The 120 bp ssODN below

tccttgcagatccacctccgctcccacactggagagagacccttcGCCTGCTCTGTCTGTGCCCATCGcttcaccaccaagggcaa**T**ctcaaggtgcactttcaccgacatccccaggtg

carried a single base mutation in the PAM sequence (in bold font) to prevent further targeting of the gene locus. A coding mutation for G416A (GGT → GCC) is underlined. The modified pool was allowed to recover, during which time genomic DNA was prepared (Sigma-Aldrich GenElute kit G1N70) and targeted integration of the mutations was confirmed by next-generation sequencing (NGS) targeted-amplicon methods. Briefly, PCR primers containing Illumina adapters were designed flanking the coding mutations and PAM mutation and used to amplify the genomic region. This PCR product was then barcoded and used for Illumina sequencing by synthesis on a MiSeq instrument. Data were generated as raw fastq files and aligned BAM files. BAM files were visualized using Integrative Genomics Viewer^30^ and fastq files were analyzed using Outknocker^31^. Rates of homology-directed repair were estimated to be 18% in the nucleofected pool. NGS data for SALL4 mock and G416A clones are provided as Supplementary Fig. 2.

SALL4 NGS forward primer sequence:

TCGTCGGCAGCGTCAGATGTGTATAAGAGACAGNNNNNNcactgtggcgctagacacat

SALL4 NGS reverse primer sequence:

GTCTCGTGGGCTCGGAGATGTGTATAAGAGACAGNNNNNNgccactttgtcctggaactc

### CRBN KO/KI genetic engineering

XCL-1 hiPSCs were co-transfected with a plasmid donor encoding both 5′ and 3′ AAVS homology arms, an EF1α promoter driving expression of a floxed (Lox2272) mKate2 cassette, and CRBN cDNA (with a PAM blocking mutation in the CRBN cDNA to prevent continued CRISPR-mediated modifications), together with Cas9/gRNA RNP complex targeting AAVS1 in human iPSC. The modified pool was allowed to recover for 5–7 days prior to mKate2 enrichment by FACs for positively integrated clones. Junction PCR, followed by Sanger sequencing, was performed on gDNA from the pools to confirm proper integration of the plasmid donor. For endogenous gene knock out of CRBN, the modified exogenous CRBN (cDNA) pool (described above) was co-transfected with Cas9/gRNA RNP complex targeting endogenous CBRN in human iPSC, together with CRE recombinase for the excision of the mKate2, thus allowing expression of the exogenous CRBN cDNA in combination with endogenous CRBN KO. Cells were allowed to recover and FACS sorted for mKate2 negative cells. Single-cell cloning was performed, and the isolates were screened by NGS as detailed above for indels in the targeted CRBN locus. NGS data confirming the lack of indels in the mock clone and the 98.6% indel frequency of the CRBN KO clone. Junction PCR confirming expression of the human CRBN gene containing the E377V/V388I double point mutation. Next-generation sequencing and junction PCR data for the CRBN KO/KI clone can be found in Supplementary Fig. 1.

5′ junction forward: CCTGAGTCCGGACCACTTTG

5′ junction reverse: ACCTAGAAGGTCCATTAGCTGC

3′ junction forward: CAGCTACGTGAATGGGATGA

3′ junction reverse: AAAAGGCAGCCTGGTAGACA

CRBN ex1 NGS forward primer sequence:

TCGTCGGCAGCGTCAGATGTGTATAAGAGACAGNNNNNNCCCTCCCTCGGAGTCTTC

CRBN ex1 NGS reverse primer sequence:

GTCTCGTGGGCTCGGAGATGTGTATAAGAGACAGNNNNNNAACAGAGCAGCGAAGAAAGC

### Single-cell cloning

Single-cell cloning for SALL4 and CRBN modified pools was performed using the ALS CellCelector single cell and colony picking platform instrument. Briefly, hiPSCs were seeded into a 6-well dish at low density and mono-clonal colonies were observed by brightfield scanning 5–7 days later. Colonies of desired diameter (500–1000 µm) were picked and re-plated into a 96-well plate. Following expansion, the plate was replicated into 2 matched 96 well plates for expansion and DNA isolation, respectively. Genetic testing of clones was performed by NGS or Junction PCR methods.

### Karyotyping analysis

XCL-1 clones were submitted to a commercial service for karyotyping (Cell Line Genetics). Standard G-banding analysis was performed on live cells that were shipped to the vendor on ice. To further interrogate the hiPSC karyotype, copy number variation testing was performed with a commercial vendor (Thermo Fisher Karyostat Analysis). Briefly, hiPSCs were grown to 70–80% confluence, passaged, pelleted, and frozen at −80 °C before sending to the vendor for analysis.

### Pluripotency analysis by FACS

Cells were dissociated into a single cell suspension, washed and fixed for 10 minutes using 4% paraformaldehyde. Cell concentration was adjusted to 1 × 10^6^ cells/mL and blocked with 3% normal goat serum with 0.1% triton-X in 1X PBS (no Ca/Mg) for 30 minutes at room temperature. Block was removed and properly diluted conjugated primary antibodies (OCT4 – AlexaFluor 488 Millipore, cat# FCMAB113A4; SSEA4 – AlexaFluor 488 StemCell Technologies, cat# 60062AD; and TRA-1–60 – Vio488 Miltenyi Biotec, cat# 130-106-872), along with unstained and isotype controls in block were added to samples and incubated overnight at 4 °C. The next day, samples were washed three times in PBS-T, re-suspended in FACs buffer and counterstained with DAPI prior to analysis on MACSQuant instrument. Data analysis was performed using FlowJo software.

### Pluripotency analysis by qRT-PCR

The TaqMan hPSC Scorecard Assay (Thermo Fisher) was used to assess the pluripotency of cultured Gibco hiPSCs and the XCL-1 hiPSC clones as described^32^. The assay outputs scores for self-renewal, ectoderm, endoderm, and mesoderm genes.

### Dose-response characterization of XCL-1 clones with the SOX17 definitive endoderm differentiation assay

XCL-1 hiPSC clones were grown in mTeSR-1 on Matrigel, passaged, counted, and seeded at ~10,000 cells/well (30,000 cells/cm^2^, ~15% confluence) in 100 µL mTeSR1 in the presence of ROCK inhibitor on Matrigel-coated µClear® 96-well plates (Greiner) for 1 d under normal culture conditions. The assay was carried out as previously described with continuous dosing of chemical for 3 total days of differentiation^23^. SOX17 staining and DAPI counter-staining were carried out as described^23^, and assays were imaged via high content imaging.

### Characterization of LPM differentiation by qRT-PCR and next generation sequencing

Gibco hiPSCs were seeded in small clumps at ~10% confluence on Matrigel-coated dishes and were cultured for 1 d in mTeSR-1. On the following day after seeding, the medium was switched to APEL2 basal medium (Stem Cell Technologies) supplemented with 5 µM CHIR99021 (Stem Cell Technologies) (hereafter referred to as APEL2 + GSKi). hiPSCs were cultured for the described duration in APEL2 + GSKi and were characterized by qRT-PCR and immunofluorescence. Briefly, RNA was extracted using buffer RLT supplemented with 1% β-mercaptoethanol and purified from hiPSCs using RNeasy kit (Qiagen) with optional DNase step. Reverse transcription was carried out using the high capacity cDNA reverse transcription kit (ThermoFisher). cDNA was characterized using Qubit HS dsDNA kit (Invitrogen), and qRT-PCR was carried out using TaqMan primer/probes (Thermo Fisher) and fast advanced master mix (Thermo Fisher) per manufacturer’s instructions on either the Viia7 or QuantStudio 5 (Thermo Fisher). Assay results were normalized to ACTB housekeeping control and the undifferentiated control (day 0) via the 2^−ΔΔCT^ method. The capacity of Gibco hiPSCs and XCL-1 hiPSCs to undergo LPM differentiation was also assessed using qRT-PCR for markers of pluripotency (*NANOG*, *POU5F1*), primitive streak (*T*, *SOX17*), LPM (*FOXF1*, *GATA4*), and limb mesenchyme (*PITX1*, *TBX5*).

Next generation sequencing was performed using Ion Torrent Ampliseq and associated reagents (Thermo Fisher). Gibco hiPSCs were seeded on Matrigel in mTeSR1 and subsequently differentiated in APEL2 + GSKi for either 1, 2, or 3 d. At each time point of differentiation, RNA was isolated by washing cells in DPBS and lysing hiPSCs with buffer RLT (Qiagen) supplemented with 1% β-mercaptoethanol (Sigma). RNA purification was performed using RNeasy kit (Qiagen) with on-column DNase treatment. Purified RNA was stored in DI water at −20 °C. Immediately prior to library prep, RNA was characterized by Qubit HS RNA kit (Invitrogen). Libraries for sequencing were prepared using the Ion AmpliSeq Transcriptome Human Gene Expression Kit (Thermo Fisher) and the Ion Ampliseq Kit for Chef DL8 (Thermo Fisher) following the manufacturer’s instructions. Assuming a library concentration after library prep of 100 pM, the library was diluted to 50 pM with molecular biology grade DI water (Qiagen). Templating was performed using the Ion 540 Kit-Chef (Thermo Fisher). Sequencing was performed on the Ion GeneStudio S5 with default settings (500 flows), and alignment was performed using hg19 AmpliSeq Transcriptome ERCC v1. Data was quantified using the AmpliSeq plugin (Thermo Fisher), and CHP files were analyzed for log_2_ expression and differential gene expression using the Transcriptome Analysis Console (TAC) software (Thermo Fisher). Data was collected for 2 independent experiments of hiPSCs undergoing LPM differentiation. The complete normalized dataset is provided as Supplementary Table 4.

### Characterization of LPM differentiation with high content imaging

hiPSCs (Gibco or XCL-1 clones) were grown in mTeSR-1 on Matrigel, passaged into small clumps with Versene (4.5 min under normal culture conditions), counted as a single-cell suspension as described above, and seeded as small clumps at approximately 10,000 cells/well (30,000 cells/cm^2^, ~15% confluence) in 100 µL mTeSR1 on Matrigel-coated µClear® 96-well plates (Greiner) for 1 d under normal culture conditions. On the day after seeding, the medium was replaced with 100 µL/well of mTeSR-1 (control) or APEL2 + GSKi containing either DMSO or diluted compounds (for a final DMSO concentration of 0.2%). The same conditions were replenished the following day for a total of 2 d of differentiation, and the following day (3 d after seeding), the medium was aspirated, and hiPSCs were fixed in 10% NBF for 15 min at room temperature. For time-course experiments, cells were fixed at the specified intervals after the start of LPM differentiation. The chemicals used in this study, atRA (Sigma), SB431542 (Stem Cell Technologies), CHIR99021, thalidomide (Celgene), pomalidomide (Celgene), and lenalidomide (Celgene) were all dissolved as stock solutions in DMSO and diluted to the described concentrations, to a final DMSO concentration of 0.1%.

Immunofluorescence staining was carried out by fixing/permeabilizing cells overnight in blocking buffer containing 3% BSA (Sigma) in DPBS-T, which contains DPBS + Ca^2+/^/Mg^2+^ with 0.3% Triton-X100 (Sigma). Fixed and permeabilized cells were incubated in primary antibodies diluted (as noted in Table 1) in antibody buffer, consisting of 1% BSA in DPBS-T, overnight at 4 °C.

**Table 1:** Primary and secondary antibody pairs used for immunofluorescence staining of hiPSCs.

Primary Antibody	Dilute	Secondary Antibody	Dilute
Goat anti-FOXF1 (R&D Systems AF4798)	1:500	Donkey anti-Goat IgG AF488 (Abcam ab150129)	1:400
Mouse anti-NANOG (Abcam ab14959)	1:200	Donkey anti-Mouse IgG AF647 (Abcam ab150107)	1:400
Mouse anti-SALL4 (Santa Cruz Clone EE-30)	1:250	Donkey anti-Mouse IgG AF647	1:400
Rat Anti-Oct4 (ThermoFisher Clone EM92)	1:250	Donkey Anti-Rat IgG DyLight 550 (Thermo SA5-10027)	1:1000
Rabbit Anti-T (Abcam Clone EPR18113)	1:500	Donkey Anti-Rabbit IgG AF790 (Thermo A11374)	1:1000

Cells were washed in blocking buffer for >6 hours at 4 °C and subsequently incubated in secondary antibody diluted in antibody buffer overnight at 4 °C. Secondary antibodies were washed out in antibody buffer for >4 h at 4 °C, and hiPSCs were counterstained with DAPI (Fisher) at 10 µg/mL in DPBS-T for 2 h. hiPSCs were washed in DPBS-T for >2 h at 4 °C, and immediately before imaging, the DPBS-T was replaced with DPBS. Initial characterization of LPM differentiation by immunofluorescence was carried out using the A1R confocal microscope (Nikon) equipped with 10X objective, and automated object analysis was carried out using NIS Elements v5 software (Nikon). Chemical dose-response experiments and experiments examining the time-course of marker expression during LPM differentiation were imaged at 10X magnification using the Cellomics ArrayScan or CellInsight Cx7 LZR High Content Screening Platform and HCS Studio Cell Analysis Software (Thermo Fisher). An artificial threshold was uniformly applied to all images to generate the percentage of DAPI+ nuclei that stained positively for FOXF1 or NANOG. Thresholding for FOXF1 and T was performed relative to undiffer-entiated hiPSCs, and thresholding for SALL4 and OCT4 was performed relative to a non-expressing cell type (human primary endothelial cells). Settings for image acquisition and processing were uniformly applied between XCL-1 clones. The capacity of all XCL-1 clones and Gibco hiPSCs to undergo LPM differentiation in the high content format was assessed by FOXF1 and NANOG immunostaining. For chemical dose responses, each mutant XCL-1 clone and its corresponding mock control were seeded on a single plate, with 8 wells per group for the APEL2 + GSKi DMSO control and the mTeSR-1 control and a single well per condition for each chemical treatment. Experiments were repeated in quadruplicate unless otherwise stated, and data are presented as mean ± SEM. Nonlinear regression analysis was performed using the 4-parameter [inhibitor]-vs-response curve fitting algorithm in Prism (GraphPad).

### 3D encapsulation of hiPSCs and characterization by qRT-PCR and DMMB assay

For 3D differentiation studies, Gibco hiPSCs were encapsulated in fibrin at 2–4 × 10^6^ cells/mL. hiPSCs were passaged with Versene (as described above) and were suspended as small clumps in a solution composed of a 1:4:5:10 dilution of 200 mM CaCl_2_ in DI water, 50 U/mL human thrombin (Sigma) in 0.1% cell culture BSA in DI water, DPBS, and hiPSC suspension in mTeSR1. The hiPSC suspension was mixed by pipetting, and 0.5 µL droplets of suspended hiPSCs were plated at the bottom of each well of a round-bottom sterile polypropylene plate. hiPSC suspensions were subsequently diluted 5-fold by pipetting up and down in each well with 2 µL of 10 mg/mL human fibrinogen (Sigma) in DPBS (sterile filtered prior to use). Fibrin clot formation proceeded for 10 minutes at room temperature prior to addition of mTeSR-1 and incubation under normal culture conditions overnight. The following day after seeding, the medium was replaced with APEL2 + GSKi. For chondrogenic differentiation experiments, cells were cultured in APEL2 + GSKi for the described duration, followed by culture in ACF MesenCult chondrogenic differentiation medium (Stem Cell Technologies) for 17–20 days under normal culture conditions. Encapsulated hiPSCs were characterized by qRT-PCR as described above, except cells were lysed by bead-based homogenization (Bead Mill 4, Thermo Fisher) in buffer RLT that was supplemented with 1% β-mercaptoethanol. RNeasy procedure was carried out per manufacturer’s instructions, including the optional DNase step. Purified RNA samples were stored at −20 °C before analysis using Taqman primer/probes (Thermo Fisher) and fast advanced master mix (Thermo Fisher) on a QuantStudio 5 (Thermo). Samples were compared using normalization to the ACTB in each sample and the control for each experiment using the 2^−ΔΔCT^ method.

Alternatively, encapsulated hiPSCs were characterized for sulfated glycosaminoglycan (sGAG) content using the dimethylmethylene blue (DMMB) assay protocol^33,34^. Briefly, 3D cultures of hiPSCs were washed in DPBS and incubated at 60 °C overnight in digestion buffer containing 5 mM L-cysteine (Sigma), 5 mM EDTA (Sigma), 100 mM sodium phosphate dibasic (Fisher Scientific), and 125 µg/mL papain (Sigma). Following digestion, samples were centrifuged at 17,000 x g for 10 min, and the supernatant was subjected to either DMMB assay for sGAG content or dsDNA analysis using the Qubit HS dsDNA assay (Thermo). The DMMB assay was performed by diluting 20 µL of sample in 200 µL DMMB assay buffer containing 9.5 mM acetic acid (Sigma), 40 mM glycine (Sigma), 27 mM sodium chloride (Sigma), and 38 µM DMMB (Sigma) in DI H_2_O. After addition of DMMB assay buffer, samples were immediately read at 525 nm, and sGAG composition was calculated in each sample by comparison to a chondroitin sulfate standard that was dissolved in digestion buffer. sGAG content (in µg/mL) was normalized to dsDNA content (in µg/mL) in each sample.

### Quality control

The hiPSCs used here were assessed for quality by karyotyping, qRT-PCR, and immunostaining. Standard G-banding karyotype analysis revealed that the XCL-1 clones exhibited an apparently normal karyotype, as defined by ≥19/20 cells with a 46,XY karyotype, however the CRBN mock clone 2A5 exhibited an undetermined karyotype (16/20 46,XY karyotype). Copy number variation analysis revealed an apparently normal karyotype for the Gibco hiPSCs and CRBN mock, SALL4 mock, and SALL4^G416A^ clones. However, the CRBN KO/KI clone exhibited an apparently aberrant karyotype with a deletion at region p.15.33 of chromosome 5 and a gain in copy number at the q35 and q36.1 regions of chromosome 7. The authors acknowledge that the apparently aberrant karyotypes of the CRBN mock and KO/KI clones may complicate the interpretation of the results from those clones. hiPSCs were, however, characterized for pluripotency to establish their usefulness in screening. All five hiPSC lines used here were characterized by the qRT-PCR based hPSC Pluripotency Scorecard and were determined to be pluripotent based on the software plugin (Supplementary Table 1). XCL-1 clones were characterized for pluripotency by flow cytometry. All XCL-1 clones were positive for OCT4 (>99% in all clones), TRA-1–60 (>99% in all clones), and SSEA4 (>98% in all clones) relative to IgG1 or IgG3 control (Supplementary Fig. 3). These data together demonstrate a sufficient degree of pluripotency of the XCL-1 clones for implementation in screening.

## Results

### Characterization of hiPSC lateral plate mesoderm differentiation by qRT-PCR and immunofluorescence

**Figure 1:**
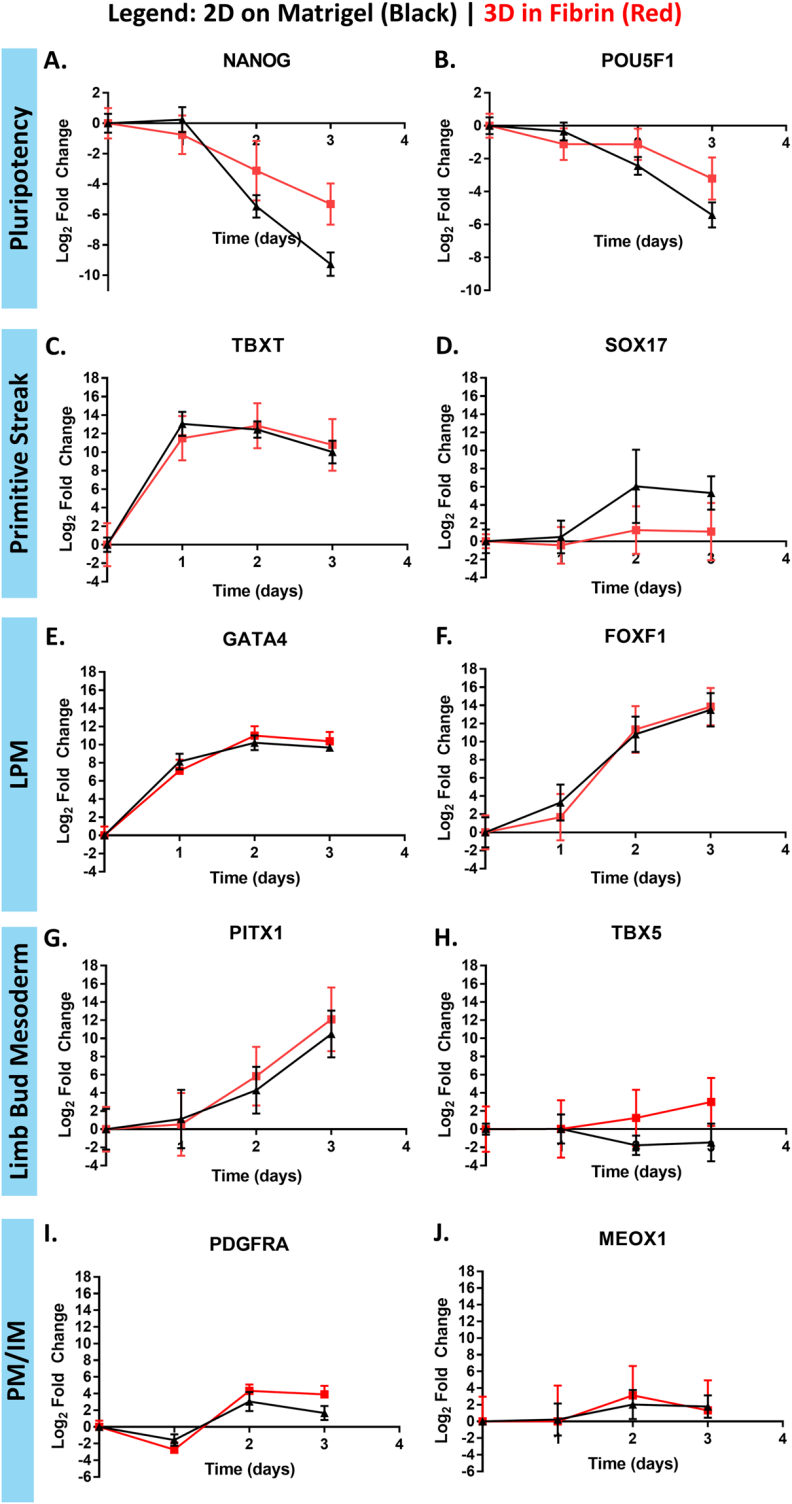
qRT-PCR characterization of the time-course of lateral plate mesoderm differentiation. Gibco hiPSCs were cultured either in 2D on Matrigel (black triangles) or 3D in fibrin (red squares) in APEL2 + GSKi for the indicated duration, and samples were quantitated with qRT-PCR. Data is presented as mean ± SEM of the log2 fold change in expression relative to the ACTB housekeeping control and the day 0 control, aggregated from at 4 independent experiments. Data represent the time-course of relative expression of pluripotency genes *NANOG* (**A**), and *POU5F1* (**B**), primitive streak genes *TBXT* (**C**) and *SOX17* (**D**), lateral plate mesoderm genes *GATA4* (**E**) and *FOXF1* (**F**), limb bud mesenchyme genes *PITX1* (**G**) and *TBX5* (**H**), the paraxial mesoderm gene *PDGFRA* (**I**), and the intermediate mesoderm gene *MEOX1* (**J**).

Directed differentiation of hiPSCs cultured in APEL2 + GSKi was characterized by time-dependent changes in markers consistent with specification to LPM. The hiPSCs used here were first characterized for pluripotency (Supplementary Table 1, Supplementary Fig. 3). The LPM differentiation assay developed here was adapted from literature describing the derivation of LPM-like cells from mouse and human pluripotent stem cells via small molecule inhibition of GSK3^27,28^. Culture of unmodified Gibco hiPSCs in APEL2 + GSKi containing 5 µM CHIR99021, a small molecule inhibitor of GSK3β^35^, for 1d was associated with increased expression of mesendoderm marker brachyury (*TBXT, T*) (Fig. 1C) and *GATA4* (Fig. 1E), a putative LPM differentiation marker^36^, and to a lesser extent, *FOXF1* (Fig. 1F), a bona fide LPM marker, relative to the undifferentiated control^24,37^. Relative to 1 d, culture in APEL2 + GSKi for 2 d was associated with reduced expression of pluripotency markers *NANOG* (Fig. 1A) and *POU5F1* (Fig. 1B), maintenance of *TBXT* expression, and significantly increased expression of mesendoderm markers *GATA4* and *SOX17* (Fig. 1D) and LPM markers *FOXF1* and *PITX1* (Fig. 1G), which is known to be expressed in the developing hind limb of mice^24^. Culture in APEL2 + GSKi for 3 d was associated with further reduction in *NANOG* and *POU5F1* expression, maintenance of the expression of *TBXT*, *SOX17*, and *GATA4*, and further increased expression of *FOXF1* and *PITX1*, relative to 2 d. No change in expression was observed for the other measured gene markers of limb mesenchyme, *TBX5* (Fig. 1H), paraxial mesoderm, *PDGFRA* (Fig. 1I), and intermediate mesoderm, *MEOX1* (Fig. 1J), compared to the undifferentiated control. Using the pluripotency scorecard panel, the most highly expressed genes in the hiPSCs differentiated for 2 d in APEL2 + GSKi were the mesendoderm gene *EOMES* and the mesoderm genes *HOPX*, *FOXF1*, and *CDX2* (Supplementary Table 3). Based on the role of FOXF1 in the specification of LPM *in vivo*^37^, FOXF1 was used as a marker of LPM differentiation for screening.

**Figure 2:**
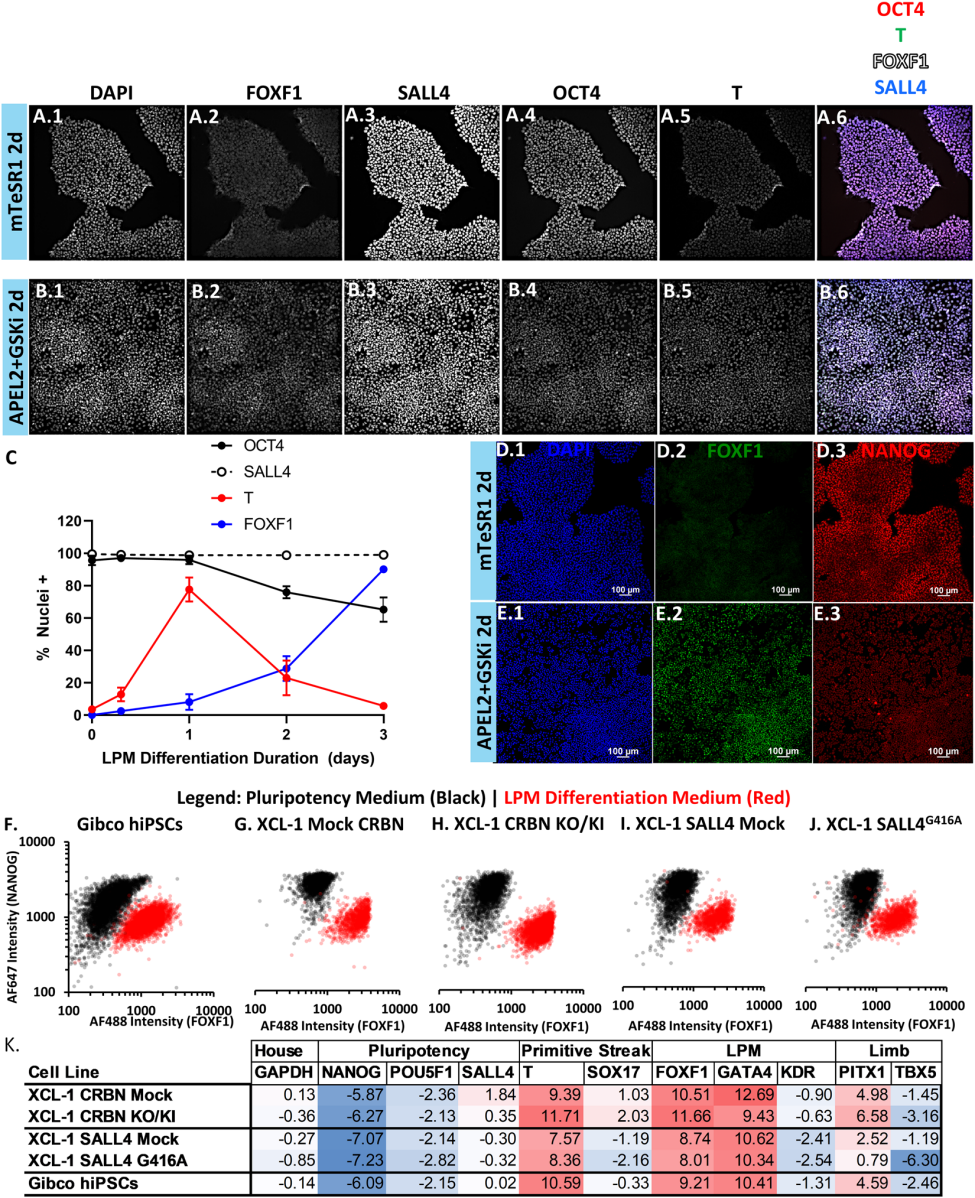
Characterization of Gibco and XCL-1 hiPSCs Undergoing LPM Differentiation by High Content Imaging and qRT-PCR. (**A**,**B**) High content imaging-based 5-color assay of Gibco hiPSCs cultured in mTeSR1 (**A**) versus APEL2 + GSKi (**B**) for 2 days. hiPSCs were co-stained for DAPI (panel 1), FOXF1 (panel 2), SALL4 (panel 3), OCT4 (panel 4), and T (panel 5). Panel 6 contains a false-colored merged image of OCT4 (red), T (green), FOXF1 (white) and SALL4 (blue) expression. (**C**) Quantification of marker expression in Gibco hiPSCs by high content imaging, presented as the mean ± SEM aggregated from 3 independent experiments of the % nuclei expressing each marker. Thresholding for each marker was performed relative to a non-expressing control cell type (human endothelial cells) for SALL4 and OCT4 or relative to undifferentiated hiPSCs for FOXF1 and T. (**D**,**E**) Confocal laser scanning microscopy images of Gibco hiPSCs cultured in mTeSR1 (**D**) versus APEL2 + GSKi (**E**) conditions for 2d and co-stained for DAPI (panel 1), FOXF1 (panel 2), and NANOG (panel 3). (**F**–**J**) Scatter plot of FOXF1 and NANOG intensity of DAPI + objects tabulated from cultures of Gibco hiPSCs (**F**), XCL-1 CRBN Mock hiPSCs (**G**), XCL-1 CRBN KO/KI hiPSCs (**H**), XCL-1 SALL4 Mock hiPSCs (**I**), and XCL-1 SALL4^G416A^ hiPSCs (**J**) that were cultured either in mTeSR1 (black circles) or APEL2 + GSKi (red circles) and co-stained for DAPI, FOXF1 (x-axis), and NANOG (y-axis). **(K)** qRT-PCR characterization of Gibco hiPSCs and XCL-1 hiPSCs cultured in APEL2 + GSKi for 2 days relative to the undifferentiated control for each cell type cultured in mTeSR1. Data are presented as the log2 fold change relative to *ACTB* and undifferentiated control for pluripotency markers *NANOG*, *POU5F1*, and *SALL4*, primitive streak genes *TBXT* and *SOX17*, LPM genes *FOXF1*, *GATA4*, and *KDR*, and limb mesoderm genes *PITX1* and *TBX5*. Color scales denote increased expression (red) or decreased expression (blue) relative to undifferentiated control.

**Figure 3:**
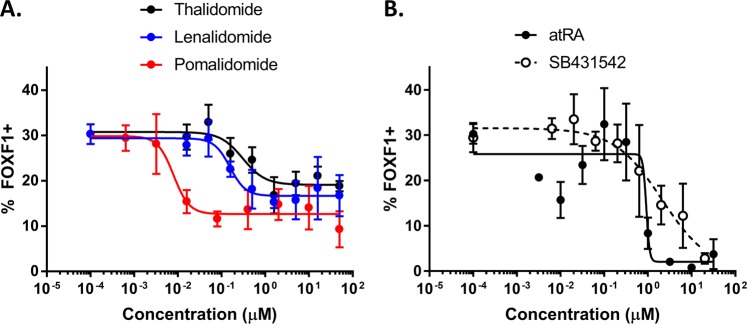
Dose-response characterization of the influence of thalidomide, lenalidomide, pomalidomide, atRA, and SB431542 on LPM differentiation in Gibco hiPSCs. High content imaging analysis of Gibco hiPSCs that were differentiated for 2 days in APEL2 + GSKi in the presence of compound and subsequently stained for FOXF1 and NANOG (only FOXF1 data shown here) and counter-stained with DAPI. Untreated DMSO control is represented as the lowest concentration in in each graph. (**A**,**B**) Data represent the mean +/− SEM %FOXF1 +/DAPI+ cells from at least 4 independent experiments. Nonlinear regression analysis was performed using the 4-parameter [inhibitor] vs response variable slope plotting tool in GraphPad Prism. (**A**) Dose-response of thalidomide (black), lenalidomide (blue), and pomalidomide (red). (**B**) Dose-response of atRA (filled circles) and SB431542 (hollow circles).

A high content imaging-based assay was developed to quantify the extent of LPM differentiation over time in hiPSCs. Gibco hiPSCs were cultured either in maintenance conditions or APEL2 + GSKi and stained for markers OCT4, SALL4, T, and FOXF1. hiPSCs prior to differentiation expressed abundant OCT4 (>95%) and SALL4 (>99%) (Fig. 2A,C). hiPSCs undergoing LPM differentiation maintained expression of SALL4 throughout differentiation and lost expression of OCT4 after 2 d (75%) with further loss of expression after 3 d (65%) of culture in APEL2 + GSKi (Fig. 2B,C). Upon differentiation in APEL2 + GSKi, hiPSCs rapidly gained expression of T after 8 h (12%) and 1 d (77%) of differentiation and thereafter lost T expression after 2 d and 3 d of differentiation (Fig. 2B,C). hiPSCs gained expression of FOXF1 after 1 d (8%) and further gained expression after 2 d (28%) of directed differentiation in APEL2 + GSKi in general agreement with qRT-PCR data, and FOXF1 expression increased to 90% of cells by 3 d of differentiation (Fig. 2C). Given that 2 d of hiPSC culture in APEL2 + GSKi drove gene and protein expression changes consistent with an LPM-like phenotype, and that 3 d of culture resulted in nearly 100% hiPSC positivity for FOXF1, subsequent studies focused on quantification of LPM differentiation after 2 d of culture to maximize the sensitivity of the assay to chemical disruption. hiPSCs abundantly expressed the pluripotency marker NANOG and were negative for FOXF1 expression upon culture in mTeSR1 (Fig. 2D) and lost NANOG expression and gained FOXF1 expression (Fig. 2E) upon culture in APEL2 + GSKi for 2 d. Quantification of NANOG and FOXF1 nuclear co-staining in Gibco hiPSCs (Fig. 2F) and XCL-1 hiPSCs (Fig. 2G–J) cultured in mTeSR1 versus APEL2 + GSKi for 2 d revealed a similar reduction in NANOG staining and increase in FOXF1 staining upon LPM differentiation. Gibco and XCL-1 hiPSCs also exhibited a similar fold decrease in *NANOG* and *POU5F1* expression and increase in *TBXT*, *FOXF1*, and *GATA4* expression upon differentiation in APEL2 + GSKi (Fig. 2K). All XCL-1 lines exhibited robust induction of FOXF1 nuclear colocalization (<2% in mTeSR1 versus >29% in APEL2 + GSKi) and reduction of NANOG nuclear colocalization (>75% in mTeSR1 versus <10% in APEL2 + GSKi) upon differentiation in APEL2 + GSKi for 2 d compared to hiPSCs maintained in mTeSR1 *via* high content imaging. The %FOXF1 + hiPSCs upon differentiation in APEL2 + GSKi was statistically indistinguishable between XCL-1 clones (Supplementary Fig. 4). The hiPSCs studied here therefore possessed a similar capacity to undergo LPM differentiation in APEL2 + GSKi. Using Gibco hiPSCs and FOXF1 as a marker for LPM differentiation, the LPM-like hiPSC differentiation assay was sensitive to inhibition by thalidomide, lenalidomide, and pomalidomide (Table 2, Supplementary Fig. 5). Pomalidomide (0.01 µM IC_50_) exerted the most potent inhibitory effect on % FOXF1 + hiPSCs, followed by lenalidomide (0.16 µM IC_50_) and thalidomide (0.31 µM IC_50_) (Fig. 3A). The hiPSC LPM-like differentiation assay also exhibited sensitivity to the known limb teratogen atRA (0.82 µM IC_50_) and TGFβ inhibitor SB431542 (2.76 µM IC_50_) (Fig. 3B), both of which also potently inhibited hPSC definitive endoderm differentiation *in vitro*^23^.

**Table 2:** IC_50_ Values (±SEM) for chemical treatments in LPM differentiation assay.

hiPSC Line	Gibco hiPSCs	XCL-1 hiPSC			
Clone	N/A	CRBN Mock	CRBN KO/KI	SALL4 Mock	SALL4^G416A^
Thalidomide	0.31 ± 0.19	0.56 ± 0.45	>50	0.69 ± 0.50	>50
Pomalidomide	0.01 ± 0.01	0.02 ± 0.01	>50	0.09 ± 0.09	>50
Lenalidomide	0.16 ± 0.08	0.09 ± 0.08	>50	0.84 ± 1.2	>50
atRA	0.82 ± 0.78	2.70 ± 2.0	8.17 ± 5.1	2.94 ± 1.2	9.10 ± 5.9
SB431542	2.76 ± 5.58	0.65 ± 0.18	0.79 ± 0.4	0.63 ± 0.17	0.51 ± 0.10

### Influence of compounds on LPM differentiation of wild-type and genetically modified hiPSCs

To determine if thalidomide- and IMiD-induced inhibition of hiPSC mesendoderm differentiation is mediated by CRBN and SALL4, hiPSC clones were genetically engineered to express either CRBN E377V/V388I mutant or SALL4 G416A mutant. Using CRISPR/Cas9-mediated gene editing, both *CRBN* alleles were knocked out in XCL-1 hiPSCs cells, followed by the insertion at the *AAVS1* locus of a transgene encoding human *CRBN* with E377V and V388I mutations (clone hereafter referred to as CRBN KO/KI). The CRBN KO/KI clone was characterized with next-generation sequencing to confirm efficient knockout of endogenous *CRBN* and with junction PCR to confirm expression of the inserted mutant human *CRBN*^*E377V/V388I*^ transgene (Supplementary Fig. 1). In addition, XCL-1 hiPSCs were engineered with CRISPR/Cas9 to express a homozygous SALL4 G416A mutation (hereafter referred to as the SALL4^G416A^ clone), and the homozygous SALL4 G416A mutation was confirmed by next-generation sequencing (Supplementary Fig. 2).

**Figure 4:**
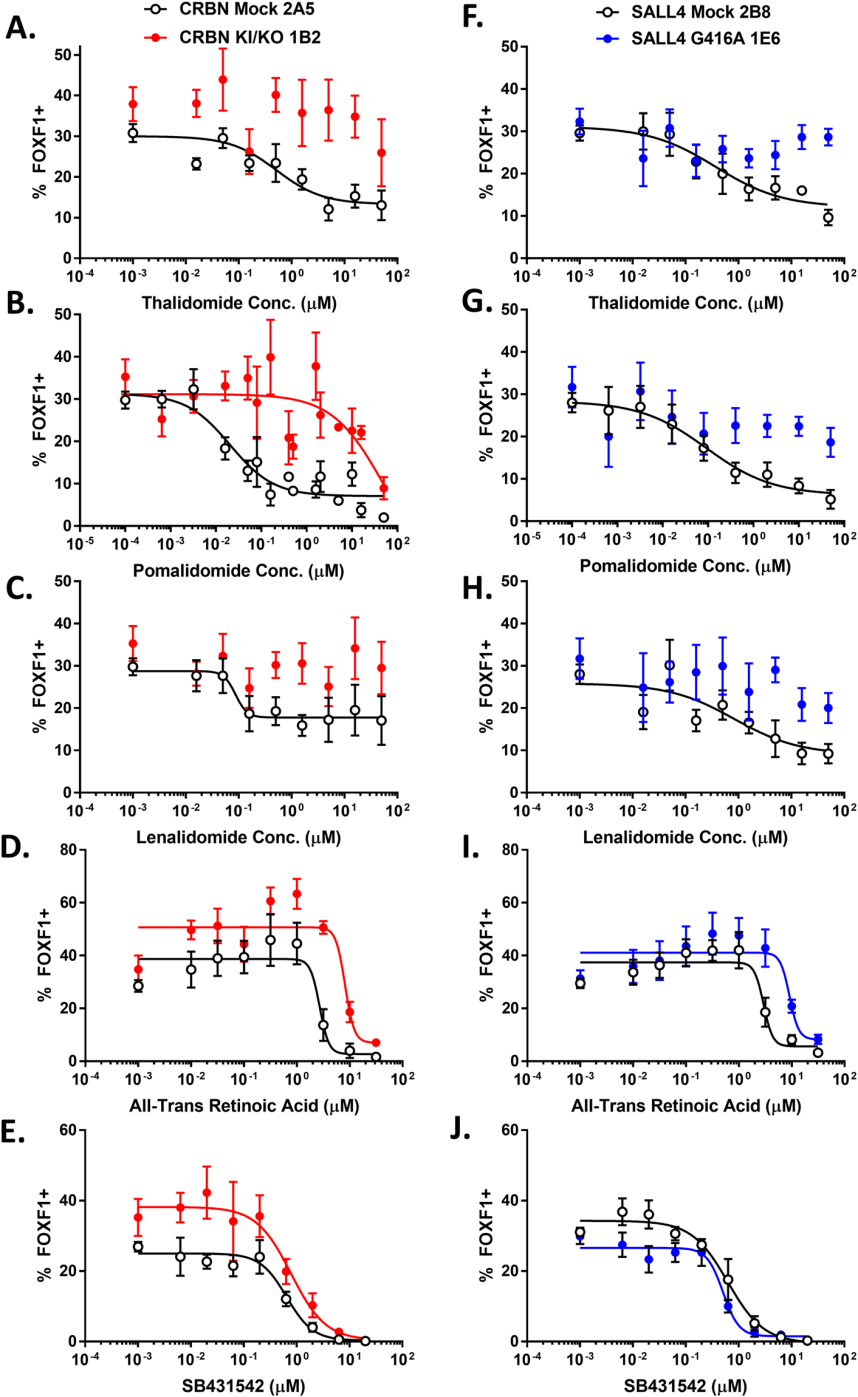
Dose-response characterization of the influence of thalidomide, lenalidomide, pomalidomide, atRA, and SB431542 on LPM differentiation of XCL-1 clones. High content imaging analysis of XCL-1 hiPSC clones that were differentiated for 2 days in APEL2 + GSKi in the presence of compound and subsequently stained for FOXF1 and NANOG (only FOXF1 data shown here) and counter-stained with DAPI. Untreated DMSO control is represented as the lowest concentration in each graph. Data represent the mean +/− SEM %FOXF1+/DAPI+ cells from at least 4 independent experiments. Nonlinear regression analysis was performed using the 4-parameter [inhibitor] vs response variable slope plotting tool in GraphPad Prism. (**A**–**E**) Dose-response characterization of thalidomide (**A**), pomalidomide (**B**), lenalidomide (**C**), atRA (**D**), and SB431542 (**E**) in CRBN mock (hollow circles) or CRBN KO/KI (red filled circles) clones. (**F**–**J**) Dose-response characterization of thalidomide (**F**), pomalidomide (**G**), lenalidomide (**H**), atRA (**I**), and SB431542 (**J**) in SALL4 mock (hollow circles) and SALL4^G416A^ (blue filled circles) clones.

Control XCL-1 hiPSC clones (CRBN mock and SALL4 mock) were sensitive to the inhibitory effect of thalidomide, pomalidomide, and lenalidomide on LPM differentiation (Table 2). Pomalidomide was the most potent inhibitor of LPM differentiation in CRBN mock (0.02 µM IC_50_) (Fig. 4B) and SALL4 mock clones (0.09 µM IC_50_) (Fig. 4G), compared to the inhibitory effect of thalidomide (0.56 and 0.69 µM IC_50_ for CRBN mock (Fig. 4A) and SALL4 mock (Fig. 4F) clones, respectively) and lenalidomide (0.09 and 0.84 µM IC_50_ for CRBN mock (Fig. 4C) and SALL4 mock (Fig. 4H) clones, respectively). The CRBN KO/KI clone was insensitive to inhibition by either thalidomide, pomalidomide, or lenalidomide (>50 µM IC_50_) (Fig. 4A–C). However, atRA (Fig. 4D) and the TGFβ inhibitor SB431542 (Fig. 4E) inhibited LPM differentiation of the mock CRBN and CRBN KO/KI clones to a similar extent, with mock and CRBN KO/KI clones exhibiting IC_50_ of 2.7 and 8.17 µM, respectively, for atRA and 0.65 and 0.79 µM, respectively, for SB431542. The SALL4^G416A^ mutant hiPSC clone, similarly to the CRBN KO/KI clone, was insensitive to inhibition by thalidomide (Fig. 4F), pomalidomide (Fig. 4G), and lenalidomide (Fig. 4H) in the LPM differentiation assay (>50 µM IC_50_). Both the SALL4 mock control and the SALL4^G416A^ clones were sensitive to inhibition by atRA (IC_50_ of 2.94 µM and 9.1 µM, respectively) (Fig. 4I) and SB431542 (IC_50_ of 0.63 µM and 0.51 µM, respectively) (Fig. 4J). The right-shift in potency of atRA in both engineered clones (~3-fold) was lower in magnitude than the right-shift in potency of thalidomide, lenalidomide, and pomalidomide (>50-fold). Compound treatment did not dose-dependently influence cell number after LPM differentiation (Supplementary Fig. 6). Therefore, compound effects on FOXF1 expression and thus on LPM differentiation were likely independent of a non-specific cytotoxic effect. Unlike unmodified hiPSCs, CRBN KO/KI and SALL4^G416A^ hiPSCs were insensitive to the effects of thalidomide, lenalidomide, and pomalidomide on LPM differentiation but retained similar sensitivity to atRA and SB431542.

### Influence of compounds on definitive endoderm differentiation of wild-type and genetically engineered hiPSCs

The XCL-1 hiPSC clones were assessed for sensitivity to thalidomide and IMiD drugs using a SOX17 definitive endoderm differentiation assay that accurately predicts teratogenicity of thalidomide^23^. The SOX17 definitive endoderm differentiation assay with mock hiPSC clones was sensitive to inhibition by thalidomide and pomalidomide, while the genetically engineered hiPSCs (CRBN KO/KI and SALL4^G416A^) were refractory to the inhibitory effect of thalidomide, lenalidomide, and pomalidomide (IC_50_ > 50 µM) (Table 3, Supplementary Fig. 7). Thalidomide and pomalidomide inhibited definitive endoderm differentiation and LPM differentiation via similar CRBN and SALL4 dependence, while only the LPM differentiation assay was sensitive to inhibition by lenalidomide.

**Table 3:** IC_50_ Values ( ± SEM) for chemical treatments in SOX17 differentiation assay.

hiPSC Line	XCL-1 hiPSC			
Clone	CRBN Mock	CRBN KO/KI	SALL4 Mock	SALL4^G416A^
Thalidomide	1.9 ± 2.3	41 ± 37	1.8 ± 2.6	>50
Pomalidomide	0.02 ± 0.02	>50	0.01 ± 0.01	>50
Lenalidomide	33 ± 21	>50	44 ± 77	>50

### Influence of compounds on SALL4 protein expression by high content imaging

**Figure 5:**
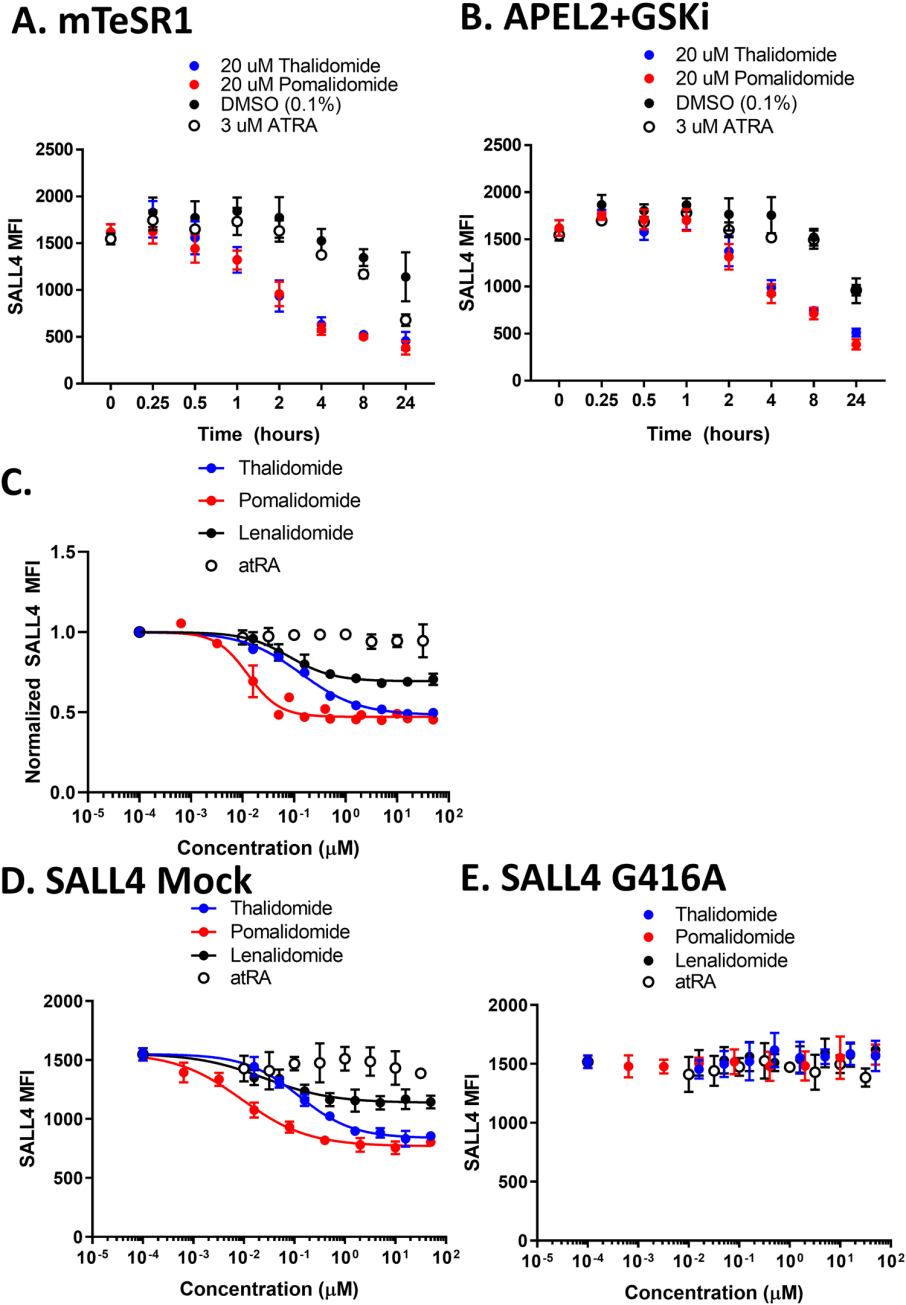
Characterization of SALL4 Degradation in hiPSCs by High Content Imaging. High content imaging analysis of SALL4 immunofluorescence with DAPI counterstaining in hiPSCs. (**A**,**B**) Time-course of SALL4 mean fluorescence intensity (MFI) in Gibco hiPSCs that were treated for the indicated time with 20 µM thalidomide (blue circles), 20 µM pomalidomide (red circles), 3 µM atRA (open circles), or DMSO (black circles) in mTeSR1 (**A**) or APEL2 + GSKi (**B**). (**C**) Dose-response characterization of thalidomide (blue circles), pomalidomide (red circles), lenalidomide (black circles), or atRA (open circles) on SALL4 MFI (normalized to the DMSO control) of Gibco hiPSCs that were differentiated in APEL2 + GSKi for 8 h in the presence of chemical. (**D**,**E**) Dose-response characterization of thalidomide (blue circles), pomalidomide (red circles), lenalidomide (black circles), or atRA (open circles) on SALL4 MFI of SALL4 mock (**D**) and SALL4^G416A^ (**E**) XCL-1 hiPSCs that were differentiated in APEL2+ GSKi for 8 h in the presence of chemical. Data represent the mean +/− SEM from 2 independent experiments. Nonlinear regression analysis was performed using the 4-parameter [inhibitor] vs response variable slope plotting tool in GraphPad Prism.

SALL4 nuclear expression was rapidly and potently reduced upon thalidomide and IMiD treatment in hiPSCs. Unmodified Gibco hiPSCs were either differentiated in APEL2 + GSKi or maintained in mTeSR1, and SALL4 nuclear protein expression was monitored by high content imaging and reported as mean fluorescence intensity (MFI). Treatment either with 20 µM thalidomide or 20 µM pomalidomide led to a rapid decline in SALL4 MFI 2 h after dosing that remained low at 8 and 24 h after dosing either in mTeSR1 (Fig. 5A) or APEL2 + GSKi (Fig. 5B). Treatment with thalidomide (0.14 ± 0.02 µM IC_50_), lenalidomide (0.08 ± 0.02 µM IC_50_), or pomalidomide (0.013 ± 0.002 µM IC_50_) reduced SALL4 MFI after 8 h of treatment (Fig. 5C). The magnitude of the decrease in SALL4 MFI in hiPSCs was most pronounced with pomalidomide (~55% decrease) and least pronounced with lenalidomide (~29% decrease). Treatment with atRA did not reduce SALL4 MFI (Fig. 5C). Further, thalidomide, lenalidomide, and pomalidomide treatment potently reduced SALL4 MFI in SALL4 mock control hiPSCs after 8 h of treatment (Fig. 5D), while the SALL4 MFI in the SALL4^G416A^ hiPSC clone was unchanged upon treatment with either thalidomide, lenalidomide, pomalidomide, or atRA (Fig. 5E).

### qRT-PCR characterization of the influence of thalidomide and atRA on LPM differentiation

**Figure 6:**
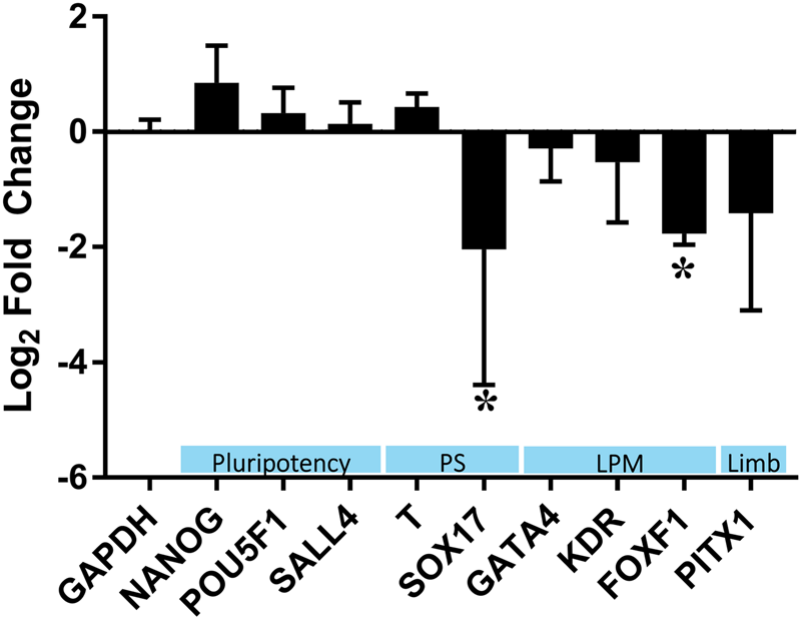
qRT-PCR characterization of the influence of 20 µM thalidomide on hiPSC LPM differentiation. Gibco hiPSCs were differentiated in APEL2 + GSKi for 2 days in the presence of 20 µM thalidomide or 0.1% DMSO. Data represent the mean ± log2 fold change, normalized to the ACTB control and the DMSO control via the 2^−ΔΔCT^ method, relative to DMSO control from 4 independent experiments. Asterisks denote statistical significance via 2-tailed Student’s t-test relative to a nominal mean log2 fold change of ‘0’ (α = 0.05). PS = primitive streak; LPM = lateral plate mesoderm.

Gene expression analysis was used to assess the effect of thalidomide on LPM differentiation and investigate whether thalidomide broadly arrested hiPSC differentiation. Although the magnitude of the elevation in *FOXF1* transcript expression was greater than that of *SOX17* during LPM differentiation, thalidomide treatment significantly reduced the expression of both *FOXF1* and *SOX17* during LPM differentiation. Thalidomide did not affect expression of the other measured markers of pluripotency (*NANOG*, *POU5F1*), primitive streak (*TBXT*), LPM (*GATA4*, *KDR*), or limb bud (*PITX1*) (Fig. 6). *SALL4* transcript expression was unchanged throughout LPM differentiation (Supplementary Fig. 8) with or without thalidomide treatment (Fig. 6), which is consistent with evidence that thalidomide induces proteasomal degradation of SALL4^10^. Treatment with thalidomide reduced expression of mesoderm-specific genes and modestly increased expression of self-renewal genes. Among the panel of 92 genes evaluated, the expression of *SOX17* was most affected by thalidomide exposure (>95% reduction), and *FOXF1* expression was reduced by ~60% (Supplementary Table 3). Further experiments are needed to understand the transcriptional changes that occur downstream of thalidomide-induced degradation of SALL4 and mis-regulation of factors involved in limb development^1^.

### A 3D hiPSC culture model to assess chondrogenic differentiation potential of LPM-like cells

**Figure 7:**
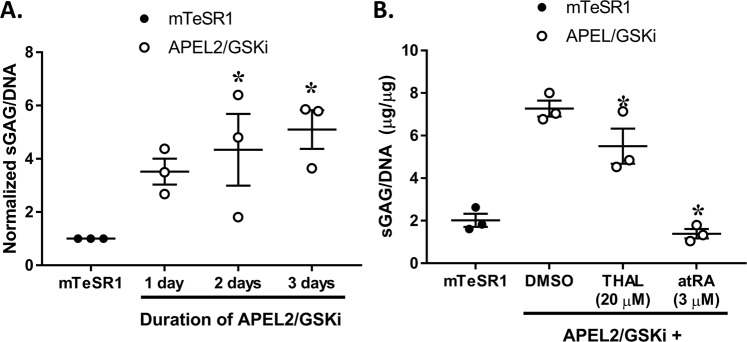
Characterization of hiPSC chondrogenic differentiation in 3D by DMMB assay for sulfated glycosaminoglycans. Chondrogenic differentiation of hiPSCs was assessed by culturing hiPSCs in fibrin gels followed by culture in APEL2/GSKi and subsequent culture in chondrogenic differentiation medium for a total of 21 days in culture after encapsulation. The influence of the duration of LPM differentiation or of chemical treatment during LPM differentiation (for 2 days) on chondrogenic differentiation was assessed by sulfated glycosaminoglycan assay on day 21. (**A**) Mean ± SEM sGAG/DNA, normalized to the mTeSR1 control for each experiment, representing 3 independent experiments (mean of each experiment plotted as a separate point) comparing the hiPSCs that were cultured in APEL2/GSKi for 1, 2 or 3d followed by 19, 18, or 17 d of chondrogenic differentiation, respectively. Asterisks denote statistically significant increase in normalized sGAG/DNA relative to the mTeSR1 undifferentiated control via one-way ANOVA and Tukey’s post-hoc test at α = 0.05. (**B**) Mean ± SEM sGAG content per DNA content from each condition from 3 independent experiments (mean of each experiment plotted as a separate point) wherein the hiPSCs were cultured in APEL2/GSKi for 2 d in the presence of either 0.1% DMSO, 20 µM thalidomide, or 3 µM atRA and subsequently cultured in chondrogenic differentiation medium for 18 d with no chemical treatments. Asterisks denote a statistically significant decrease relative to the APEL2/GSKi DMSO control via one-way ANOVA and Tukey’s post-hoc test at α = 0.05.

To examine the relevance of disrupted LPM differentiation to a limb-specific phenotype, LPM-like cells were differentiated along a chondrogenic lineage and assessed *via* qRT-PCR and a DMMB assay for sulfated glycosaminoglycans (sGAG), a marker of limb bud chondrogenesis^38^. LPM differentiation in fibrin-encapsulated 3D cultures of hiPSCs exhibited similar gene expression changes as differentiation in 2D culture on Matrigel (Fig. 1). Chondrogenic differentiation subsequent to LPM differentiation for 2 or 3 d induced expression of early chondrogenic markers *COL2A1* (after 7 d and 14 d) and *SOX9* (after 7 and 14 d) and late chondrogenic differentiation marker *ACAN* (after 14 and 21 d) relative to the undifferentiated control (Supplementary Fig. 9). The duration of LPM differentiation in 3D affected chondrogenic marker expression and sGAG abundance of hiPSCs. Only hiPSCs that were differentiated to the LPM lineage for 2 or 3 d, but not for 1 d, followed by chondrogenic differentiation exhibited increased expression of *SOX9* and *COL2A1* after 7 d (Supplementary Fig. 9) and increased sGAG/DNA content after 21 d (Fig. 7A) relative to the undifferentiated control. To address whether chemical treatment during LPM differentiation interfered with subsequent chondrogenic differentiation, hiPSCs in 3D were treated with DMSO or a noncytotoxic concentration (Supplementary Fig. 10) of thalidomide (20 µM) or atRA (3 µM) during LPM differentiation for 2 d prior to 18 d of chondrogenic differentiation. Treatment with atRA or thalidomide during LPM differentiation in 3D reduced *FOXF1* and *KDR* expression, both markers of LPM, as well as *SOX17* expression. atRA uniquely reduced the expression of *TBXT* and *GATA4* in hiPSCs undergoing LPM differentiation (Supplementary Fig. 11), suggesting that atRA blocked differentiation at the level of TBXT, a master regulator of mesoderm specification^39^. Cultures of hiPSCs that were treated with either thalidomide or atRA during LPM differentiation and subsequently differentiated along a chondrogenic lineage exhibited decreased sGAG/DNA content compared to the DMSO control (Fig. 7B).

## Discussion

The results here show for the first time that thalidomide, lenalidomide, and pomalidomide inhibit human stem cell mesendoderm differentiation along LPM or definitive endoderm lineage via CRBN-mediated degradation of SALL4. SALL4 has been directly implicated in limb development in mice and humans^12,13^. Two distinct differentiation assays were used to interrogate the SALL4-dependence of thalidomide and IMiD teratogenicity. Differentiation of hiPSCs along either a definitive endoderm lineage (SOX17) or LPM lineage (FOXF1) was sensitive to inhibition by thalidomide, lenalidomide, and pomalidomide. hiPSCs engineered to express human *CRBN* containing E377V and V388I point mutations (with the endogenous *CRBN* locus knocked out) were insensitive to inhibition by thalidomide, lenalidomide, or pomalidomide in either definitive endoderm or LPM differentiation assays. Thalidomide- and IMiD-induced degradation of GSPT1 is abolished by the point mutation E377V, while degradation of IKZF1, IKZF3, ZFP91, and CK1α is abolished by point mutation V388I^40^. Homozygous mutation of the endogenous mouse *Crbn* locus to the human-like *Crbn*^*I391V*^ restores the degradation of neosubstrates Ikzf1, Ikzf3, Zfp91, and Ck1α in mice^10,41^. However, human CRBN is unable to ubiquitinate mouse SALL4 in the presence of thalidomide^10^, suggesting that species sensitivity to thalidomide- and IMiD-induced teratogenicity is dependent on both CRBN and SALL4 sequence and structure. Pomalidomide, lenalidomide, and thalidomide failed to induce SALL4 degradation in human cells expressing *CRBN* with the V388I mutation relative to cells expressing wild-type human *CRBN* (Supplementary Fig. 12, uncropped gels in Supplementary Fig. 13). The CRBN KO/KI hiPSC clone was insensitive to thalidomide- and IMiD-mediated inhibition of mesendoderm differentiation in both the DE and LPM differentiation assays, though at the highest concentration tested (50 µM), thalidomide (Fig. 4A) and pomalidomide (Fig. 4B) inhibited LPM differentiation albeit not to the same magnitude as control hiPSCs. These data suggest thalidomide and pomalidomide may interfere with non-CRBN-mediated targets and thereby disrupt LPM differentiation at high concentrations. Numerous studies have hypothesized molecular targets for thalidomide that could explain CRBN-independent effects at saturating concentrations of thalidomide and pomalidomide^42^. For example, lenalidomide and pomalidomide can bind to and reduce TP53RK activity and thereby inhibit p53 phosphorylation and induce p53-mediated apoptosis in multiple myeloma cells in a CRBN-independent fashion^43^. *TP53RK* was expressed abundantly in hiPSCs here (Supplementary Table 4), although its role during LPM differentiation is unclear. However, as a mediator of p53-dependent apoptosis, TP53RK is unlikely a target of thalidomide-mediated disruption of LPM differentiation, as there were no significant effects on cell number elicited by thalidomide or pomalidomide in the LPM differentiation assay. The CRBN KO/KI hiPSC clone was insensitive to thalidomide, lenalidomide, and pomalidomide, however this insensitivity may be attributed to abrogated degradation of CRBN neosubstrate(s) besides SALL4.

To address specifically the SALL4 dependence of hiPSC mesendoderm differentiation, parental hiPSCs were engineered to express a homozygous G416A mutation in the endogenous *SALL4* locus. SALL4^G416A^ is unable to bind CRBN in the presence of thalidomide^11^, and SALL4^G416A^ is resistant to thalidomide-induced degradation mediated by CRBN in hiPSCs^10^. An hiPSC clone expressing SALL4^G416A^ was insensitive to the effects of thalidomide, pomalidomide, and lenalidomide on either definitive endoderm differentiation (SOX17) or LPM differentiation (FOXF1). Thalidomide, lenalidomide, or pomalidomide treatment during LPM differentiation rapidly and dose-dependently reduced nuclear SALL4 in contrast to atRA that interfered with LPM differentiation without any corresponding change in SALL4. All cell lines tested here were sensitive to the control teratogen atRA and the TGFβ inhibitor SB431542, showing that the engineered hiPSCs (CRBN KO/KI or SALL4^G416A^) were specifically insensitive to the effects of thalidomide, lenalidomide, and pomalidomide. Retinoic acid is an endogenous limb morphogen^44^, and excess retinoic acid exposure *in utero* produces an array of birth defects in mice that include limb malformations^45^. SB431542 is a small molecule inhibitor of TGFβ signaling through ALK4, ALK5, and ALK7 that blocks primitive streak induction in mESCs^46^. Both SB431542 and atRA^23,47^ block hPSC definitive endoderm differentiation and were shown here to disrupt LPM differentiation in a CRBN- and SALL4-independent fashion. In contrast, thalidomide, lenalidomide, and pomalidomide inhibited hiPSC mesendoderm differentiation *via* CRBN-mediated SALL4 degradation. However, the modest inhibition of LPM differentiation induced by pomalidomide (Fig. 4G) and lenalidomide (Fig. 4H) at the two highest concentrations tested in the SALL4^G416A^ clone suggests that at high concentrations, pomalidomide and lenalidomide may inhibit LPM differentiation in a CRBN-dependent but SALL4-independent fashion. Thalidomide and its analogs degrade numerous C2H2 zinc finger-containing neosubstrates via CRBN modulation^48^, and it is reasonable to suspect that a zinc finger besides SALL4 may be degraded in the presence of thalidomide and its analogs to induce limb teratogenicity. Two closely related SALL4 family members, SALL1 and SALL3, have previously been implicated in mouse limb development^49^ and are expressed abundantly in the hiPSCs used here (Supplementary Table 4). Although SALL1, SALL2, and SALL3 are predicted to bind to the pomalidomide-CRBN complex via docking simulations^48^, neither SALL1, SALL2, nor SALL3 are degraded in the presence of thalidomide, lenalidomide, or pomalidomide in human embryonic stem cells^11^, suggesting that SALL1, SALL2, and SALL3 are not neosubstrates of CRBN in the presence of thalidomide or IMiDs. Additionally, p63 has been identified as a putative neosubstrate of CRBN in the presence of thalidomide and a molecular mediator of thalidomide teratogenicity whose degradation can be induced by thalidomide in adult human epithelial cells^50^. *TP63* was not expressed in hiPSCs here (Supplementary Table 4) in agreement with Asatsuma-Okumura *et al*.^50^. Thus, although p63 may be a putative CRBN neosubstrate in the presence of thalidomide and may play a role in thalidomide teratogenicity, p63 is unlikely a mediator of thalidomide-, lenalidomide-, and pomalidomide- mediated disruption of LPM differentiation. Future work is warranted to exhaustively rule out the role of other C2H2 zinc finger proteins in thalidomide-induced teratogenicity. The data here establish a mechanistic link between a putative molecular and hypothesized cellular mechanism of thalidomide teratogenicity.

We sought here to develop an *in vitro* teratogenicity assay that recapitulates the early stem cell specification events upstream of limb bud development. The gold standard *in vitro* assay for teratogenicity testing has been the mEST, which measures the influence of putative teratogens on the spontaneous cardiac differentiation of mouse embryoid bodies^20,51–53^. The mEST presents several limitations, namely the subjectivity of analysis, and was characterized as 53–79% accurate for predicting teratogenicity depending on the chemical reference set used^16,17^. Thalidomide is teratogenic in humans, NHPs, and rabbits but does not induce teratogenicity in mice^1,7^, and indeed the mEST has been appropriately insensitive to the effects of thalidomide, though it is sensitive to the effects of other known mouse and human teratogens including atRA^20^. Recently, an hPSC-based teratogenicity assay of definitive endoderm differentiation was established and characterized as 94% predictive of visceral malformations in preclinical species^23^. The definitive endoderm differentiation assay is sensitive to thalidomide at sub-micromolar concentrations^23^, which highlights a breakthrough in *in vitro* assays to predict human teratogenicity. However, no *in vitro* teratogenicity assay to-date has been used to characterize lenalidomide or pomalidomide. Pomalidomide^54^ and lenalidomide^55^ have been shown to induce limb malformations in embryofetal development studies in rabbits and NHPs, respectively. Evidence of teratogenicity with pomalidomide and lenalidomide along with their structural similarity to thalidomide led both pomalidomide and lenalidomide to be considered probable human teratogens^54,55^. Here, a definitive endoderm differentiation assay was used in parallel with a novel LPM differentiation assay to examine the molecular mechanism whereby thalidomide, lenalidomide, and pomalidomide disrupt mesendoderm differentiation.

Pomalidomide and thalidomide inhibited hiPSC differentiation either to definitive endoderm or LPM, while the LPM differentiation assay here was uniquely sensitive to inhibition by lenalidomide. These data suggest that the LPM differentiation assay may be more predictive of lenalidomide-induced limb teratogenicity than the SOX17 definitive endoderm differentiation assay or that LPM differentiation is more dependent on SALL4 than definitive endoderm differentiation. Pomalidomide was more potent and elicited a greater magnitude of response than either thalidomide or lenalidomide in the mesendoderm differentiation assays and the SALL4 degradation assay. However, though lenalidomide induced SALL4 degradation with the weakest potency and magnitude relative to thalidomide, the inhibitory effects of lenalidomide and thalidomide in the LPM differentiation assay were similar in magnitude at saturating concentrations. The LPM differentiation assay may thus lack the sensitivity to distinguish chemicals that degrade SALL4 to varying magnitudes. The enhanced sensitivity of the LPM differentiation to lenalidomide versus thalidomide suggests that lenalidomide might induce the degradation of additional CRBN neosubstrate(s) besides SALL4, thus sensitizing the hiPSCs to the effect of SALL4 degradation on the differentiation of mesoderm (Table 2) but not definitive endoderm (Table 3). However, the lack of sensitivity of the SALL4^G416A^ hiPSC clone to the effects of thalidomide, lenalidomide, or pomalidomide on mesendoderm differentiation suggests that thalidomide, lenalidomide, and pomalidomide inhibit hiPSC differentiation in unmodified hiPSCs primarily through degradation of SALL4. The data herein demonstrate the utility of hiPSCs for modeling SALL4-independent and SALL4-dependent limb teratogens.

SALL4 plays a central role in embryonic development, specifically in the regulation of limb development^12,13^, mouse embryonic stem cell pluripotency^56^, and hematopoiesis^57^. The data here suggest that thalidomide- and IMiD-mediated degradation of SALL4 disrupts FOXF1 and SOX17 expression, suggesting FOXF1 and SOX17 may be directly or indirectly regulated by SALL4 at the transcriptional level. To-date, three studies have examined SALL4 by ChIP-Seq to identify its transcriptional targets^57–59^. Two separate studies (in mESCs^58^ and human CD34+ cells^57^) demonstrated that SALL4 can bind to the *SOX17* promoter^57,58^. In mouse extraembryonic endoderm cells, Sall4 occupies the *Gata4*, *Gata6*, and *Sox17* promoters by ChIP-Seq, and shRNA knockdown of Sall4 expression reduces the mRNA level of *Gata6*, *Gata4*, and *Sox17*^58^, demonstrating that Sall4 directly regulates the transcription of *Gata6*, *Gata4*, and *Sox17*. Although *SOX17* transcript expression was decreased in thalidomide-treated hiPSCs undergoing LPM differentiation here, *GATA4* transcript expression was unaffected, suggesting that during LPM differentiation, GATA4 may be under transcriptional control by other regulators besides SALL4. Further, *Sox17* expression during definitive endoderm differentiation is abolished in *Sall4* knockout mouse ESCs^60^. These data point to a direct interaction between SALL4 and SOX17 that agrees with the SALL4-dependent inhibition of SOX17 expression by thalidomide observed here. Comparatively little is known about transcriptional or functional connections between SALL4 and FOXF1, and only one ChIP-Seq study (out of the three mentioned above) identified that in mouse cells, Sall4 occupies the *Foxf1* promoter^59^. Conversely, ChIP-seq of mouse tissue discovered a Gli3 and Tbx5 binding site in the promoter of *Foxf1*^61^. Given that Sall4 activates Gli3 in the developing mouse limb^13^, FOXF1 may be under transcriptional control directly by SALL4 or indirectly by GLI3. However, Gli3 has also been demonstrated as a repressor of Foxf1 expression during mouse embryonic lung organogenesis^62^. *Foxf1*^−/−^ embryos also exhibit down-regulated expression of *Sall4* along with *Tcf21* and *Ptk7*^63^. These data suggest a complex gene regulatory network for FOXF1, GLI3, and SALL4. A thorough characterization of the crosstalk between SALL4 and other developmentally-relevant transcription factors is warranted.

Interference of LPM differentiation here disrupted subsequent chondrogenesis, thus establishing a plausible link between thalidomide- and IMiD-mediated degradation of SALL4 and a limb-specific phenotypic outcome. To evaluate whether disruption of LPM differentiation can disrupt subsequent chondrogenesis, hiPSCs were cultured in 3D and stepwise differentiated down the LPM lineage and chondrogenic lineage. This approach was motivated by the role of 3D culture in stimulating functional chondrogenesis compared to 2D culture^64^. 3D cultures of PSCs have historically been generated with forced aggregation. However, forced aggregation reduces hPSC pluripotency under maintenance conditions and may prime the resulting aggregates to spontaneous ectoderm differentiation^65^. Thus we employed a 3D culture system amenable to mesoderm differentiation. Fibrin encapsulation supports maintenance of hPSCs^66^, cardiac mesoderm differentiation of hPSCs^67^, and LPM differentiation and subsequent limb-regenerating capability of mESCs^26^. Fibrin encapsulation here maintained the pluripotency of hiPSCs (Supplementary Table 2) and supported LPM differentiation with similar magnitude and time-course of marker expression as 2D culture (Fig. 1). The approach to stepwise differentiate encapsulated hiPSCs to the LPM lineage subsequent to differentiation down the chondrogenic lineage is distinct from previous approaches to differentiate hPSCs first to mesenchymal stem cells^68^ or to paraxial mesoderm^69,70^ and subsequently sclerotome cells (the developmental origin of hyaline cartilage)^71^ followed by chondrogenic differentiation in 3D aggregates. The duration of LPM differentiation influenced sGAG abundance such that 2d of LPM differentiation was used for further characterization. Thalidomide (at 20 µM) or atRA (at 3 µM) treatment during LPM differentiation reduced the %FOXF1 + cells by 30% and 90%, respectively, via high content imaging, which agrees with the 30% and complete abolishment of sGAG/DNA content, respectively, upon chondrogenic differentiation in 3D. These data suggest that the relative abundance of sGAG/DNA is proportional to the relative abundance of FOXF1 + LPM-like cells and demonstrate that compromised LPM differentiation is associated with compromised chondrogenic differentiation capacity. Future work is warranted to evaluate if thalidomide- and IMiD-induced degradation of SALL4 influences limb morphogenesis in sensitive species by a direct effect on specification of limb progenitors, as suggested by the data herein, or by an indirect effect on a paracrine cell type that disrupts signaling involved in limb formation.

## Conclusions

Here we established a mechanistic link between CRBN-mediated degradation of SALL4 and teratogenicity of thalidomide, lenalidomide, and pomalidomide using a phenotypic *in vitro* human stem cell-based assay that recapitulates key aspects of limb development. The data together show that thalidomide, lenalidomide, and pomalidomide may induce limb teratogenicity by inducing degradation of SALL4 and disrupting stem cell specification to functional LPM cells. The assay described here is amenable to high content imaging, which highlights its utility for *in vitro* teratogenicity screening. The approach also highlights the utility of CRISPR-Cas9 gene editing for interrogating mechanisms of xenobiotic teratogenicity and presents a new paradigm for *in vitro* teratogenicity screening wherein chemical effects are investigated in distinct parallel differentiation assays for mesoderm or definitive endoderm. Given the dependence on SALL4, the LPM differentiation assay described here could be a useful screening tool to rapidly identify thalidomide analogs with activity against SALL4 and potential teratogenicity. Counter-screening for SALL4 degradation in an *in vitro* human developmental system is likely to be important for development of new CRBN-modulating compounds and compounds harnessing CRBN binding to induce degradation of substrates via ligand-directed degradation^72^.

## Supplementary information

Supplementary Dataset 1.

Supplementary Dataset 2.
