# Supplementary material for: Thalidomide Inhibits Human iPSC Mesendoderm Differentiation by Modulating CRBN-dependent Degradation of SALL4
Source: Sci Rep. 2020 Feb 18;10:2864. doi: 10.1038/s41598-020-59542-x (PMC7046148; doi:10.1038/s41598-020-59542-x)
Supplement: Supplementary file 1 — Supplementary Dataset 1. [file 41598_2020_59542_MOESM1_ESM.pdf]

Supplementary Information

Title: Thalidomide Inhibits Human iPSC Mesendoderm Differentiation by Modulating CRBN-dependent Degradation of SALL4

Authors: David G. Belair, Gang Lu, Laura E. Waller, Jason A. Gustin, Nathaniel Collins, Kyle L. Kolaja

<u>Table of contents:</u>

Supplementary Table 1. Pluripotency panel analysis of XCL-1 hiPSCs and Gibco hiPSCs

Supplementary Table 2. Pluripotency panel analysis of Gibco hiPSCs cultured in 2D vs 3D during maintenance vs LPM differentiation

Supplementary Table 3. Pluripotency panel results for Gibco hiPSCs cultured in mTeSR1 versus APEL2+GSKi for 2 days in the absence or presence of thalidomide

Supplementary Figure 1. Next-generation sequencing and junction PCR characterization of CRBN mock clone (2A5) and homozygous CRBN KO/KI clone (1B2).

Supplementary Figure 2. Next-generation sequencing of SALL4 mock clone (2B8) and homozygous $SALL4^{G416A}$ clone (1E6).

Supplementary Figure 3. Characterization of XCL-1 engineered clones by flow cytometry for pluripotency markers OCT4, TRA-1-60, and SSEA4.

Supplementary Figure 4. Expression of NANOG and FOXF1 by high content imaging in experiments with XCL-1 clones.

Supplementary Figure 5. Representative images of Gibco hiPSC LPM differentiation with chemical treatment.

Supplementary Figure 6. Dose-response characterization of the influence of thalidomide, pomalidomide, lenalidomide, atRA, and SB431542 on DAPI+ cell number of XCL-1 clones.

Supplementary Figure 7. Dose-response characterization of the influence of thalidomide, lenalidomide, and pomalidomide on SOX17 differentiation of XCL-1 clones.

Supplementary Figure 8. Expression of SALL4 in hiPSCs during LPM differentiation

Supplementary Figure 9. qRT-PCR data of 3D cultures of hiPSCs that were subjected to stepwise LPM differentiation followed by chondrogenic differentiation.

Supplementary Figure 10. Normalized viability of hiPSCs differentiated to LPM for 2 days in 3D culture in the presence of 20 µM thalidomide or 3 µM atRA.

Supplementary Figure 11. qRT-PCR characterization of 3D cultures of hiPSCs that were differentiated in APEL2+GSKi for 2 days in the presence of either 20 µM thalidomide or 3 µM atRA.

Supplementary Figure 12. Human CRBN E377V mutation abolished thalidomide-, lenalidomide-, and pomalidomide-induced degradation of human SALL4.

Supplementary Figure 13. Uncropped Western blot gels.

Legend for Supplementary Tables 1-3

Supplementary tables 1-3 contain qRT-PCR data obtained using the Taqman hPSC Scorecard Assay (Thermo Fisher). Lysates of Gibco and XCL-1 hiPSCs were purified to RNA using RNeasy procedure (Qiagen). Reverse transcription was performed using the cDNA high capacity reverse transcription kit (Invitrogen). dsDNA content was calculated using Qubit dsDNA HS kit (Invitrogen). cDNA was loaded into hPSC pluripotency panel pre-loaded 384 well plates, and plates were run on Viia7 using standard curve method. The hPSC scorecard analysis software (Thermo Fisher) was used to calculate the relative gene expression scores for self-renewal genes, ectoderm genes, endoderm genes, and mesoderm genes. Scores are provided from pooled lysates from replicate wells of a single experiment. Cells were cultured as described herein. The data in table 1 were generated with hiPSCs that were cultured in mTeSR1 on Matrigel. The data in table 2 were generated with Gibco hiPSCs that were cultured either on Matrigel (2D) or encapsulated in fibrin (3D) and maintained either for 3 days in mTeSR1 (maintenance) or cultured for 1 day in mTeSR1 followed by 2 days of LPM differentiation in APEL2+GSKi. The data in table 3 were generated with Gibco hiPSCs that were cultured on Matrigel either in mTeSR1 or differentiated in APEL2+GSKi for 2 days in the presence of either DMSO (control) or 20 μM thalidomide.

Supplementary Table 1. Pluripotency panel analysis of XCL-1 hiPSCs and Gibco hiPSCs

Sample Name	Score: Self-renewal	Score: Ectoderm	Score: Mesoderm	Score: Endoderm	Assay Determination
XCL-1 CRBN Mock	-0.18	-0.37	0.34	-0.87	Self-renewal
XCL-1 CRBN KO/KI	-0.13	-0.09	-0.20	-1.18	Self-renewal
XCL-1 SALL4 Mock	-0.12	-0.28	0.20	-0.85	Self-renewal
XCL-1 SALL4^{G416A}	-0.11	-0.14	0.50	-0.93	Self-renewal
Gibco hiPSC	-0.66	-1.02	0.13	-1.52	Self-renewal

Legend: Gene expression relative to the reference standard

x>1.5	Upregulated
1.0<x<=1.5	
0.5<x<=1.0	
-0.5<=x<=0.5	Comparable
-1.0<=x<-0.5	
-1.5<=x<-1.0	
x<-1.5	Downregulated

Supplementary Table 2. Pluripotency panel analysis of Gibco hiPSCs cultured in 2D vs 3D during maintenance vs LPM differentiation

Sample ID		Score			
		Self-renewal	Ectoderm	Mesoderm	Endoderm
2D	mTeSR1	-0.66	-1.02	0.13	-1.52
2D	APEL2+GSKi	-5.68	-0.60	1.65	-0.02
3D	mTeSR1	-0.34	-0.51	-0.12	-1.09
3D	APEL2+GSKi	-4.83	-0.36	2.13	-0.26

Legend: Gene expression relative to the reference standard

x>1.5	Upregulated
1.0<x<=1.5	
0.5<x<=1.0	
-0.5<=x<=0.5	Comparable
-1.0<=x<-0.5	
-1.5<=x<-1.0	
x<-1.5	Downregulated

Gene	Category	Culture Condition: mTeSR1	Culture Condition: APEL2+GSKi	Culture Condition: APEL2+GSKi + 20 μM THAL
EOMES	Endoderm	0.10	234.34	233.95
HOPX	Mesoderm	1.12	233.31	266.96
FOXF1	Mesoderm	0.16	145.68	53.06
CDX2	Mesoderm	0.02	125.18	116.09
RXRG	Endoderm	0.03	99.83	45.12
SNAI2	Mesoderm	0.64	79.16	79.39
NR5A2	Mesendoderm	162.84	62.01	108.47
TBX3	Mesoderm	0.05	23.38	10.36
SOX17	Endoderm	0.03	20.73	0.60
MYO3B	Ectoderm	0.33	16.00	13.48
SST	Endoderm	1.16	14.76	31.68
HAND1	Mesoderm	0.00	14.50	3.36
PDGFRA	Mesoderm	0.56	12.00	10.72
CDH20	Endoderm	0.40	11.78	26.88
GATA4	Endoderm	0.01	10.90	9.45
GATA6	Endoderm	0.00	7.35	6.98
T	Mesendoderm	0.00	6.70	10.15
IL6ST	Mesoderm	1.26	5.93	5.08
EN1	Ectoderm	0.11	5.82	2.94
PAX3	Ectoderm	0.30	5.42	5.35
SDC2	Ectoderm	7.45	5.19	5.53
COL2A1	Ectoderm	0.68	4.78	6.16
NR2F2	Ectoderm	0.06	4.41	10.04
HAND2	Mesoderm	0.11	3.91	0.58
FOXP2	Endoderm	0.96	3.68	23.93
ABCA4	Mesoderm	2.60	3.27	2.72
KLF5	Endoderm	0.51	3.09	1.52
TRPM8	Ectoderm	0.12	2.47	0.10
ESM1	Mesoderm	0.11	1.74	1.42
WNT1	Ectoderm	0.31	1.68	0.25
FCN3	Mesoderm	0.59	1.64	1.68
FOXA2	Endoderm	0.30	1.59	2.93
NODAL	Endoderm	0.26	1.48	1.90
BMP10	Mesoderm	0.39	1.33	0.31
NKX2-5	Mesoderm	0.08	1.23	0.10
FGF4	Mesendoderm	0.04	1.19	1.54
ZBTB16	Ectoderm	0.18	0.95	0.70
HMP19	Endoderm	0.92	0.92	0.53
PLVAP	Mesoderm	1.50	0.89	0.57
CDH5	Mesoderm	2.22	0.87	0.10
HEY1	Mesoderm	0.71	0.75	1.07
CLDN1	Endoderm	1.21	0.65	0.69
FOXA1	Endoderm	0.31	0.57	1.01
GDF3	Mesendoderm	4.71	0.46	0.70
RGS4	Mesoderm	0.61	0.45	0.47
PTHLH	Mesendoderm	0.31	0.44	0.89
TM4SF1	Mesoderm	1.63	0.41	1.81
HHEX	Endoderm	0.06	0.37	0.55
PRDM1	Endoderm	0.03	0.35	0.12
CXCL5	Self-renewal	9.91	0.31	0.41
MAP2	Ectoderm	1.77	0.27	0.43
ODAM	Mesoderm	2.98	0.25	0.28
CABP7	Endoderm	0.05	0.24	0.15
TRIM22	Self-renewal	6.77	0.21	0.41
NOS2	Ectoderm	0.18	0.20	0.12
NR2F1/NR2F2	Ectoderm	0.01	0.18	0.19
HNF4A	Endoderm	0.01	0.17	0.04
ELAVL3	Endoderm	0.74	0.16	0.11
OLFM3	Ectoderm	1.96	0.16	0.06
DMBX1	Ectoderm	0.01	0.14	0.01
NPPB	Mesendoderm	0.06	0.14	0.02
PHOX2B	Endoderm	0.19	0.13	0.15
PRKCA	Ectoderm	0.31	0.13	0.20
HNF1B	Endoderm	0.02	0.11	0.01
CPLX2	Endoderm	0.05	0.09	0.09
PAPLN	Ectoderm	0.23	0.09	0.20
ALOX15	Mesoderm	1.89	0.09	0.13
POU3F3	Endoderm	0.01	0.08	0.01
POU4F1	Ectoderm	0.12	0.08	0.17
LCK	Self-renewal	0.49	0.06	0.04
NANOG	Self-renewal	1.42	0.05	0.07
COLEC10	Mesoderm	4.54	0.05	1.25
DNMT3B	Self-renewal	0.20	0.04	0.05
LEFTY1	Endoderm	0.48	0.04	0.06
SOX1	Ectoderm	0.05	0.03	0.04
LMX1A	Ectoderm	0.02	0.02	0.02
POU5F1	Self-renewal	0.32	0.02	0.01
AFP	Endoderm	0.01	0.01	0.01
PAX6	Ectoderm	0.05	0.01	0.01
DRD4	Ectoderm	0.13	0.01	0.02
CDH9	Ectoderm	0.32	0.01	0.01
HESX1	Self-renewal	0.71	0.01	0.01
SOX2	Self-renewal	1.12	0.01	0.03
IDO1	Self-renewal	1.24	0.01	0.01
LEFTY2	Endoderm	0.06	0.00	0.00

Fold change legend

fc > 100	Upregulated
10 < fc <= 100	
2 < fc <= 10	
0.5 <= fc <= 2	Comparable
0.1 <= fc < 0.5	
0.01 <= fc <0.1	
fc < 0.01	Downregulated

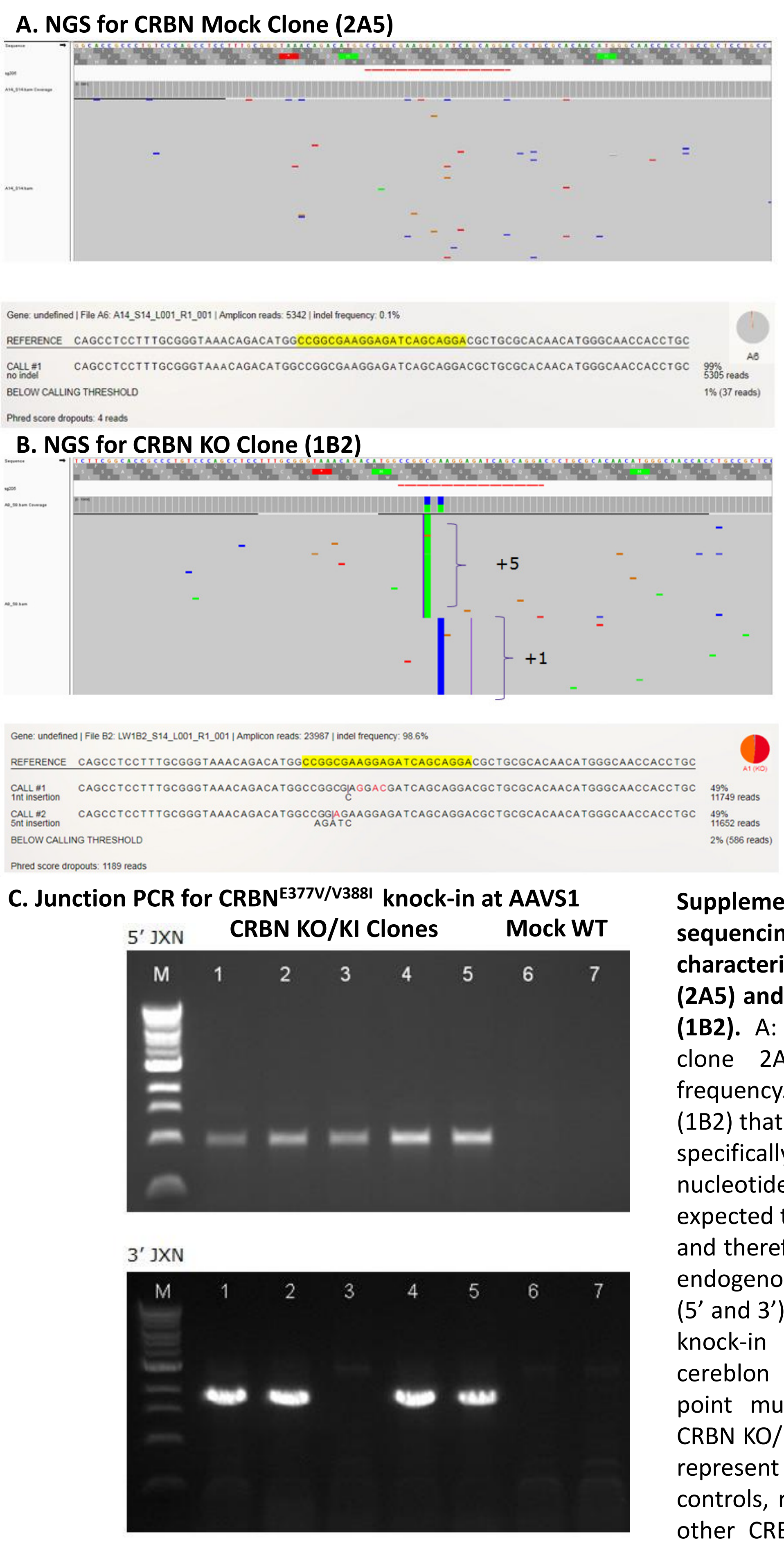

Supplementary Figure 1. Next-generation sequencing and junction PCR characterization of CRBN mock clone (2A5) and homozygous CRBN KO/KI clone (1B2). A: NGS data for the CRBN mock clone 2A5 demonstrating 0.1% indel frequency. B: NGS data for CRBN KO clone (1B2) that exhibited 98.6% indel frequency, specifically a 1 nucleotide insertion and a 5 nucleotide insertion, both of which are expected to produce a frameshift mutation and therefore a functional knockout of the endogenous CRBN locus. C: Junction PCR (5′ and 3′) to confirm the expression of the knock-in transgene encoding human cereblon with the E377V/V388I double point mutation. Lane 1 represents the CRBN KO/KI clone 1B2, while lanes 6 and 7 represent the mock (2A5) and wild-type controls, respectively. Lanes 2-5 represent other CRBN KO/KI clones that were not studied here.

A. NGS for SALL4 Mock Clone (2B8)

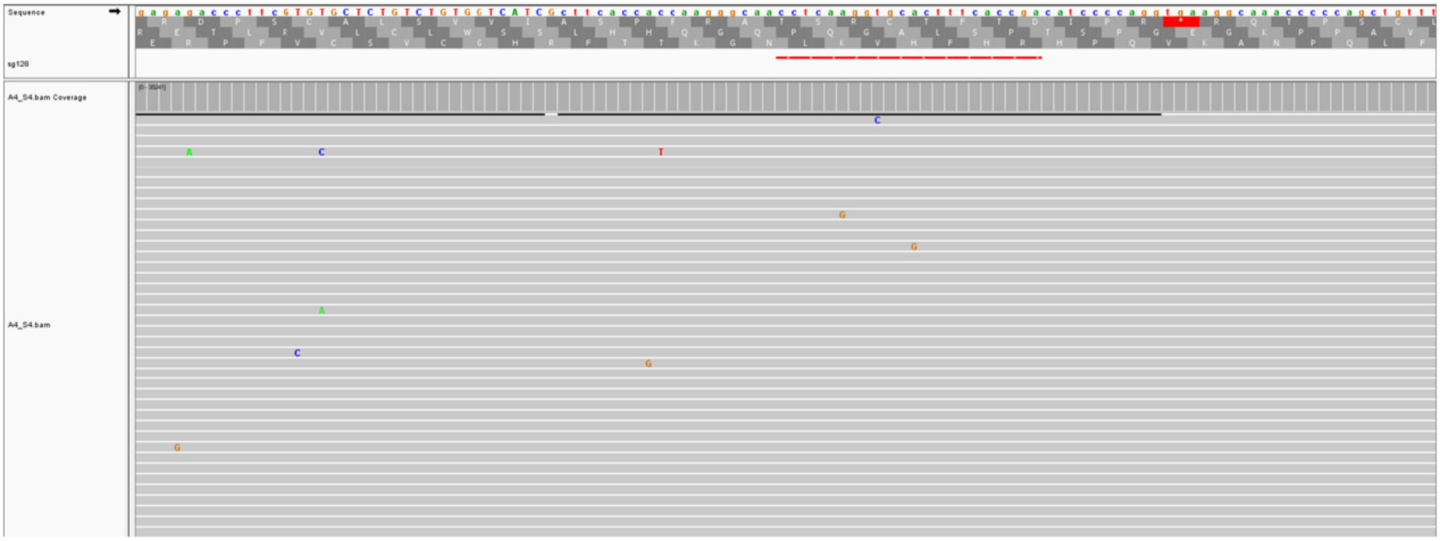

B. NGS for SALL4^{G416A} Clone (1B6)

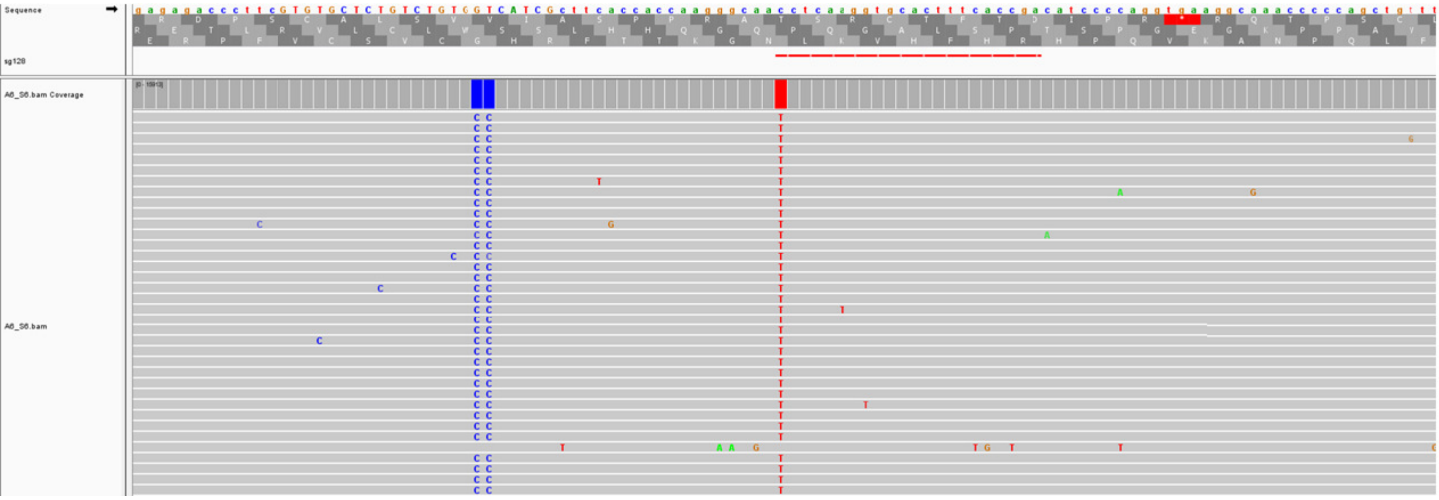

Supplementary Figure 2. Next-generation sequencing of mock clone (2B8) and homozygous SALL4^{G416A} clone (1E6). A: NGS data for the SALL4 mock clone 2B8 demonstrating no measurable indels. B: NGS data for SALL4^{G416A} clone 1E6 demonstrating the GGT to GCC mutation encoding G and A respectively. The AAC to AAC mutation still encodes N and thus does not affect the resulting SALL4^{G416A} mutant protein.

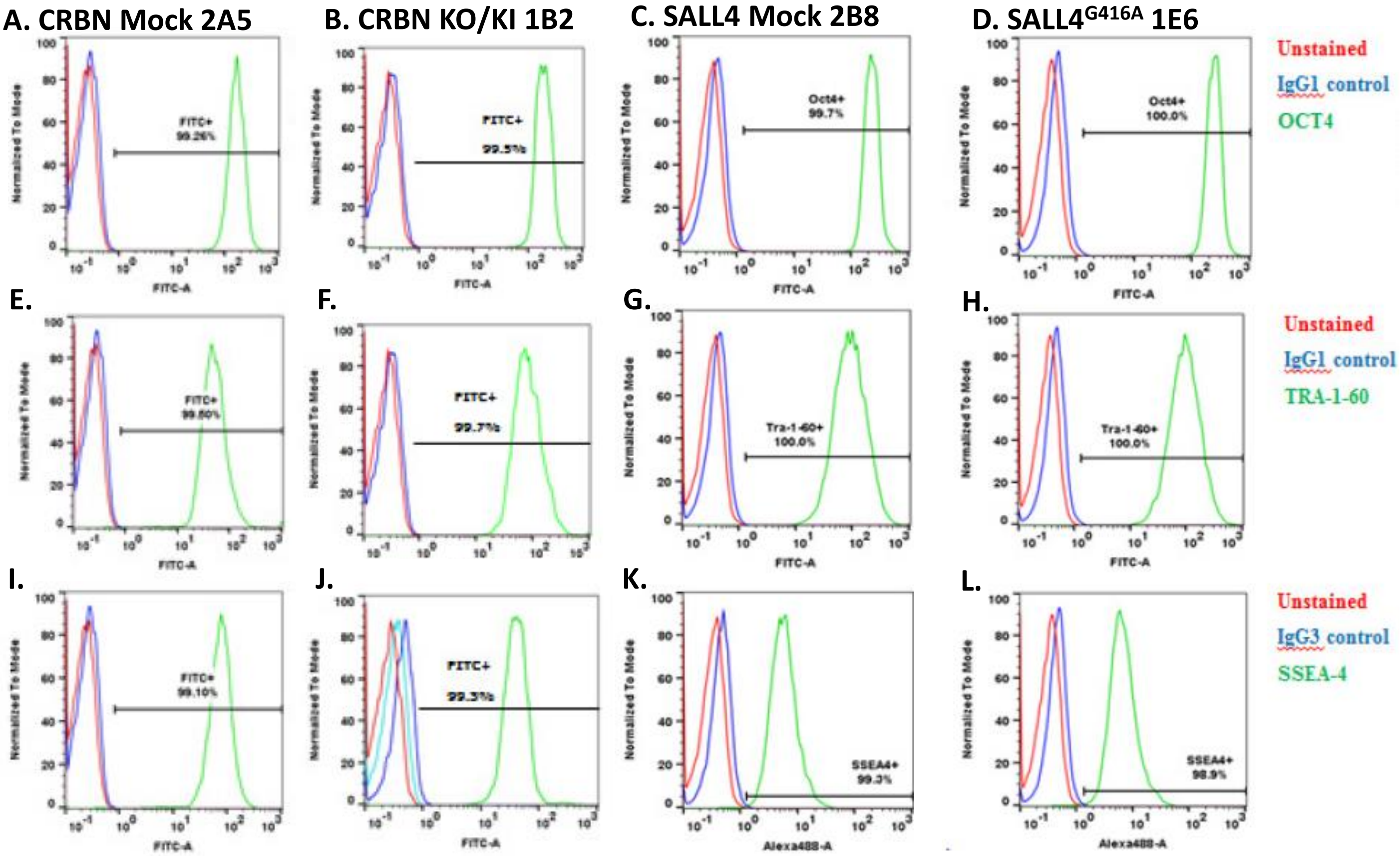

Supplementary Figure 3. **Characterization of XCL-1 engineered clones by flow cytometry for pluripotency markers OCT4, TRA-1-60, and SSEA4.** hiPSCs were characterized by flow cytometry and compared with IgG1 or IgG3 control for the expression of pluripotency markers OCT4 (A-D), TRA-1-60 (E-H), and SSEA4 (I-L) in CRBN mock clone 2A5 (A, E, I), CRBN KO/KI clone 1B2 (B, F, J), SALL4 mock clone 2B8 (C, G, K), and SALL4^{G416A} clone 1E6 (D, H, L).

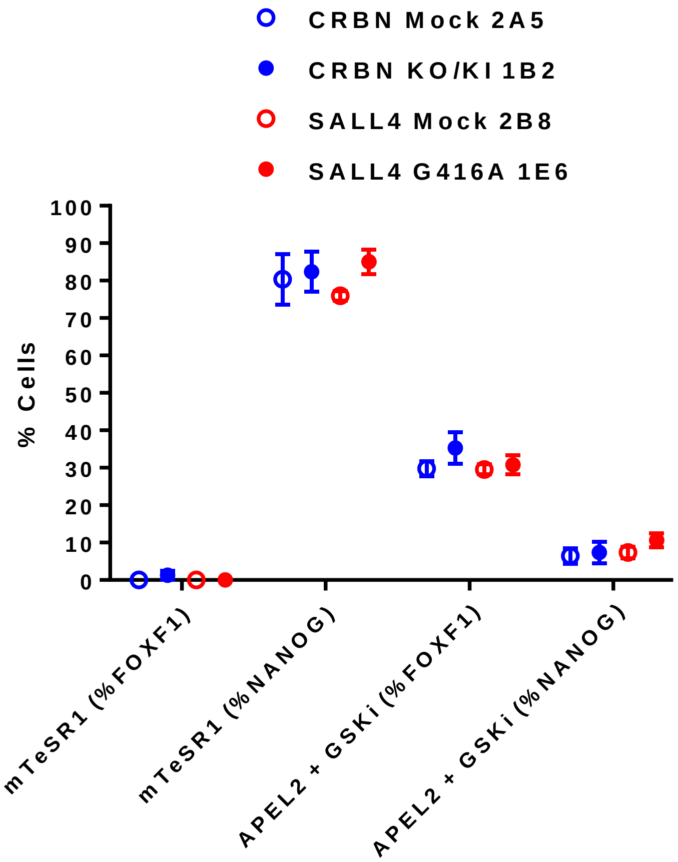

Supplementary Figure 4. Expression of NANOG and FOXF1 by high content imaging in experiments with XCL-1 clones. The relative % of cells expressing NANOG and FOXF1 were characterized by high content imaging in mTeSR1 control wells and APEL2+GSKi DMSO control wells, 8 wells per experiment. The data represent the mean ± SEM of the % DAPI+ cells expressing each marker from each group representing 5 independent experiments with the CRBN clones and 8 independent experiments with the SALL4 clones. The data points represent the CRBN mock control (empty blue circles), CRBN KO/KI clone (filled blue circles), SALL4 mock control (empty red circles), and SALL4^{G416A} clone (red filled circles). Statistical analysis revealed no significant difference in the % FOXF1+ cells or % NANOG+ cells in the APEL2+GSKi control and no significant difference in the %FOXF1 cells in the mTeSR1 control. While there was no statistical difference in the %NANOG cells in the mTeSR1 control comparing the CRBN mock and KO/KI clones, there was a statistical difference in the % NANOG cells in the SALL4 mock control versus the SALL4^{G416A} clone (two-way ANOVA and Tukey's post-hoc test at α=0.05).

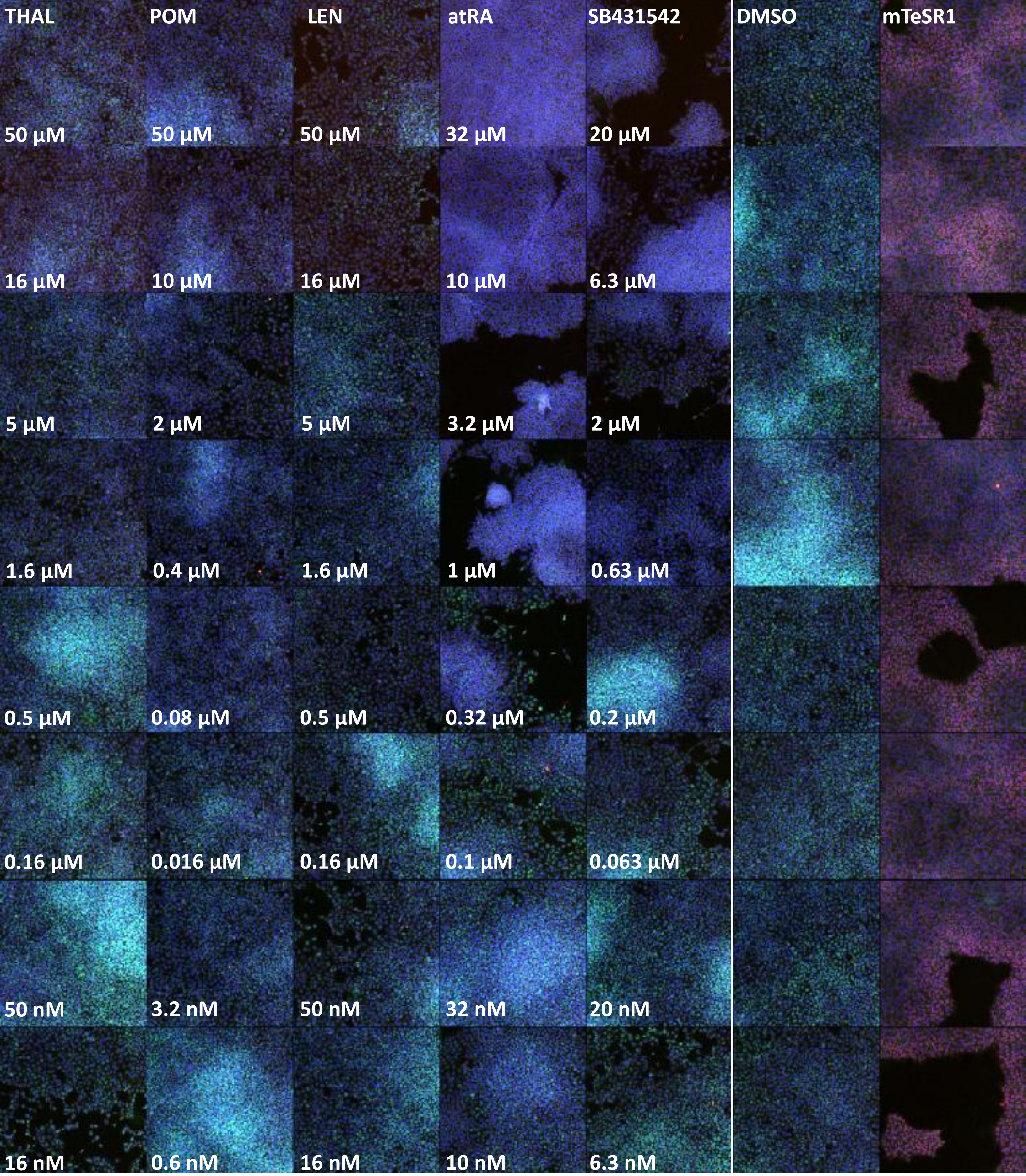

Supplementary Figure 5. Representative images of Gibco hiPSC LPM differentiation with chemical treatment. hiPSCs were cultured on Matrigel and differentiated for 2 d in APEL2+GSKi in the presence of a dose response of either thalidomide (THAL), pomalidomide (POM), lenalidomide (LEN), all-trans retinoic acid (atRA), or SB431542. The concentration represented by each 10X image is displayed. 8 replicate wells of the APEL2+GSKi DMSO control and the mTeSR1 undifferentiated control are displayed in the right two columns.

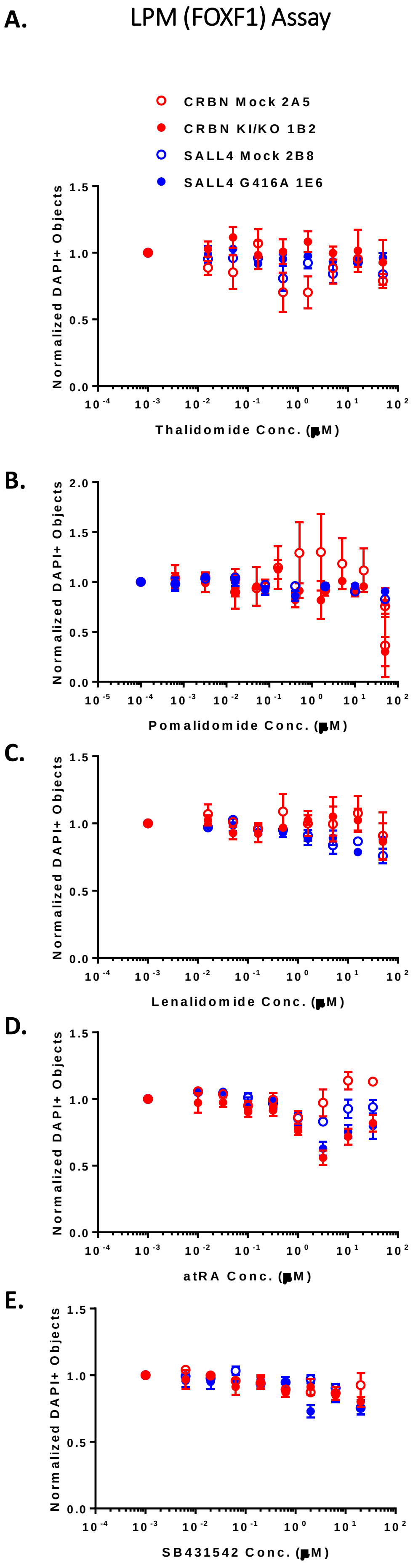

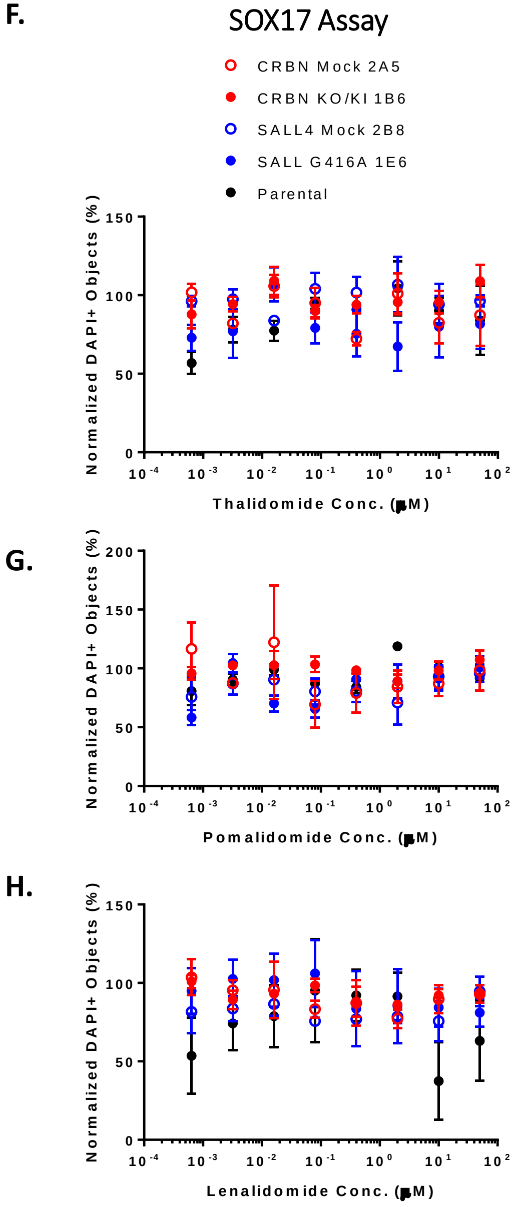

Supplementary Figure 6. Dose-response characterization of the influence of thalidomide, pomalidomide, lenalidomide, atRA, and SB431542 on DAPI+ cell number of XCL-1 clones. High content imaging analysis of XCL-1 hiPSC clones that were either differentiated to LPM (A-E) or definitive endoderm (F-H) and imaged with high content imaging. Data represent the mean ± SEM normalized number of DAPI+ objects in each condition from 4 independent experiments. A-E: Dose-response characterization of thalidomide (A), pomalidomide (B), lenalidomide (C), atRA (D), and SB431542 (E) in CRBN mock (hollow circles), CRBN KO/KI (red filled circles), SALL4 mock (hollow circles), and SALL4^{G416A} (blue filled circles) clones. F-H: Dose-response characterization of thalidomide (F), pomalidomide (G), and lenalidomide (H) in CRBN mock (hollow circles), CRBN KO/KI (red filled circles), SALL4 mock (hollow circles), and SALL4^{G416A} (blue filled circles) clones. Nonlinear regression analysis was performed using the 4-parameter [inhibitor] vs response variable slope plotting tool in GraphPad Prism. The IC_{50} values for each chemical was > 50 µM suggesting no overt cell death occurred as a result of chemical treatment through hiPSC differentiation to LPM or definitive endoderm.

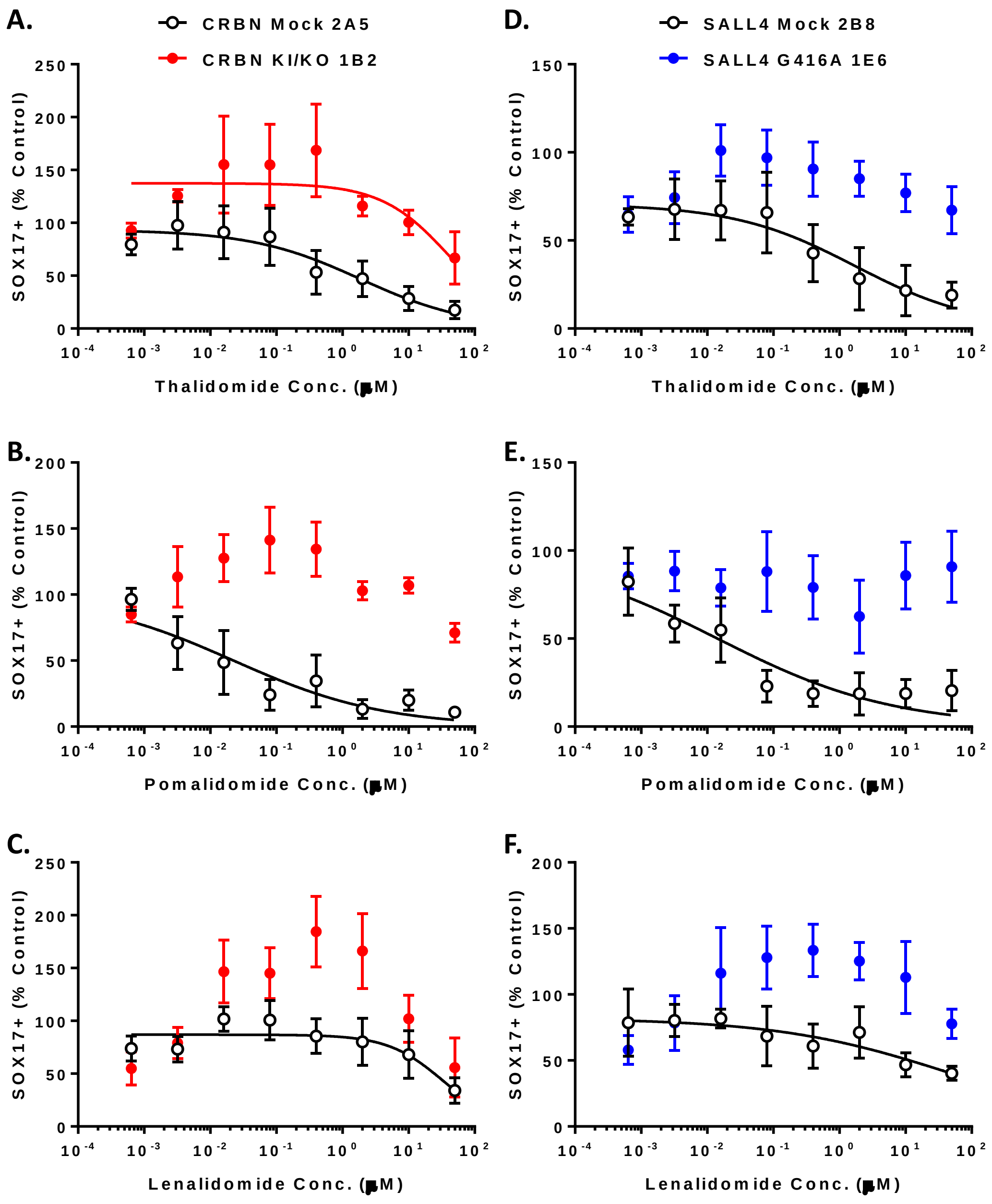

Supplementary Figure 7. Dose-response characterization of the influence of thalidomide, lenalidomide, and pomalidomide on SOX17 differentiation of XCL-1 clones. High content imaging analysis of XCL-1 hiPSC clones that were differentiated for 3 days in the presence of compound and subsequently stained for SOX17 and counter-stained with DAPI. Untreated DMSO control is omitted. Data represent the mean +/- SEM %SOX17 cells (relative to control) from 4 independent experiments. Nonlinear regression analysis was performed using the 4-parameter [inhibitor] vs response variable slope plotting tool in GraphPad Prism. A-C: Dose-response characterization of thalidomide (A), pomalidomide (B), and lenalidomide (C) in cereblon mock (hollow circles) or CRBN KO/KI (red filled circles) clones. D-F: Dose-response characterization of thalidomide (D), pomalidomide (E), and lenalidomide (F) in SALL4 mock (hollow circles) and SALL4^{G416A} (blue filled circles) clones.

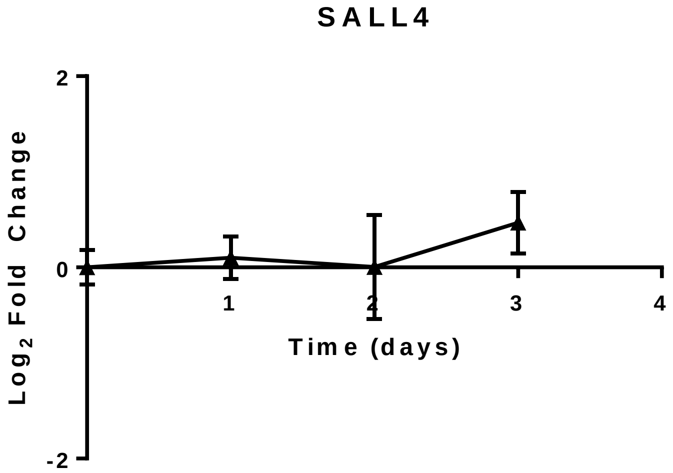

Supplementary Figure 8. Expression of SALL4 in hiPSCs during LPM differentiation. hiPSCs were cultured on Matrigel and differentiated for the ascribed duration in APEL2+GSKi. Fold change in SALL4 expression was calculated relative to the ACTB loading control and the undifferentiated control (on day 0) via the $2^{-\Delta\Delta CT}$ method. Data are presented as the mean ± SEM of the log2 fold change relative to the undifferentiated control for 6 independent experiments. No statistical difference relative to the undifferentiated control was observed with one-way ANOVA (α=0.05)

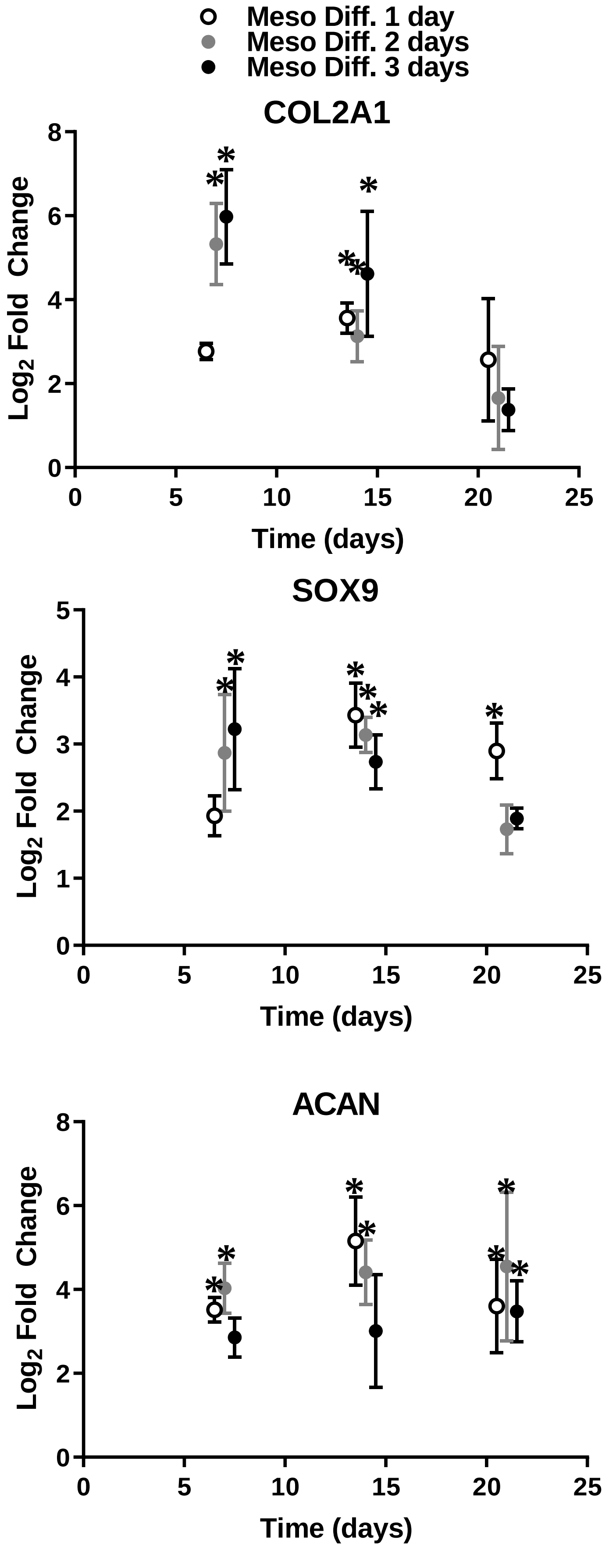

Supplementary Figure 9. qRT-PCR data of 3D cultures of hiPSCs that were subjected to stepwise LPM differentiation followed by chondrogenic differentiation. hiPSCs were encapsulated in fibrin and either differentiated for 1 (white circles), 2 (gray circles), or 3 days (black circles) in APEL2+GSKi followed by differentiation in chondrogenic differentiation until the end of the experiment at day 21. Samples were collected after 7, 14, and 21 days of total culture. All samples were double normalized to the ACTB control and mTeSR1 time-matched control using the $2^{-\Delta\Delta CT}$ method. Data represent the mean ± SD log2 fold change relative to the mTeSR1 time-matched control for 3 independent experiments. Asterisks denote statistical significance relative to the time-matched mTeSR1 control by two-way ANOVA and post-hoc Bonferroni multiple comparisons test (α=0.05).

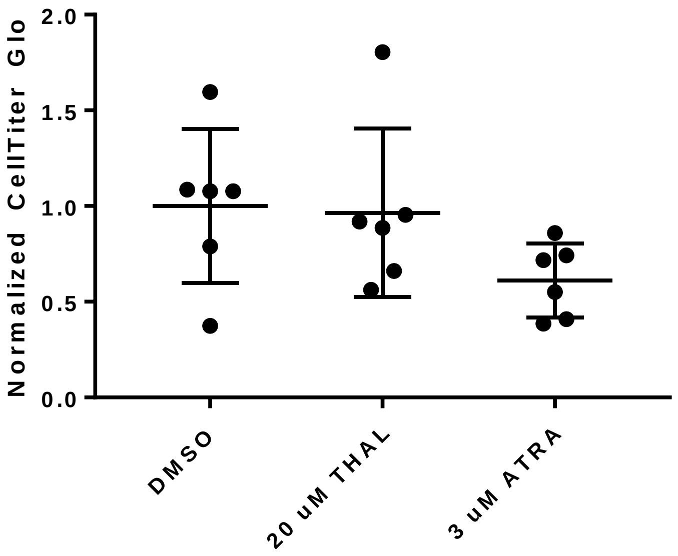

Supplementary Figure 10. Normalized viability of hiPSCs differentiated to LPM for 2 days in 3D culture in the presence of 20 µM thalidomide or 3 µM atRA. Data represent replicate wells in a single experiment, n=6, of hiPSCs that were cultured in fibrin for 1 day in mTeSR1 followed by 2 days of culture in APEL2+GSKi in the presence of either 0.1% DMSO, 20 µM thalidomide, or 3 µM atRA. Following differentiation, the medium was replaced with CellTiter Glo 3D reagent (diluted 1:1 in DPBS), and luminescence was measured on a plate reader. Normalized luminescence is reported as mean +/- SD of 6 replicate wells per condition. No statistical differences was observed via one-way ANOVA and Dunnett's post-hoc test (α=0.05).

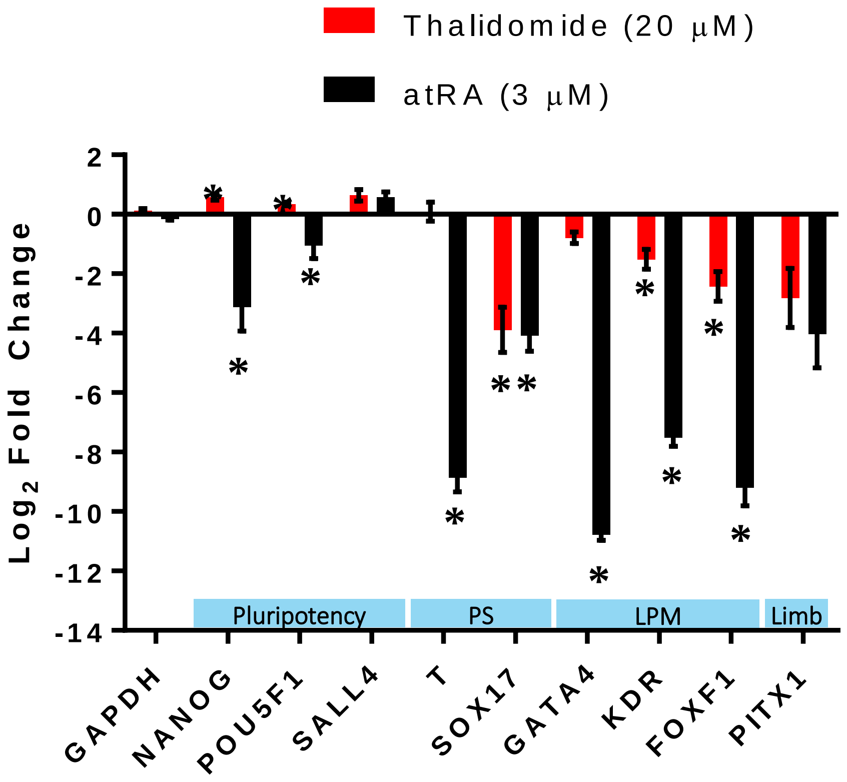

Supplementary Figure 11. qRT-PCR characterization of 3D cultures of hiPSCs that were differentiated in APEL2+GSKi for 2 days in the presence of either 20 μM thalidomide or 3 μM atRA. hiPSCs were encapsulated in fibrin and differentiated for 2 days in APEL2+GSKi in the presence of either 20 μM thalidomide (red bars) or 3 μM atRA (black bars). Data are presented as mean ± SEM of the log2 fold change calculated from each sample relative to the ACTB housekeeper control and the DMSO control (normalized via the $2^{-\Delta\Delta CT}$ method) from three independent experiments. Asterisks denote statistical significance via two-tailed t-test relative to a mean value of '0' (α=0.05).

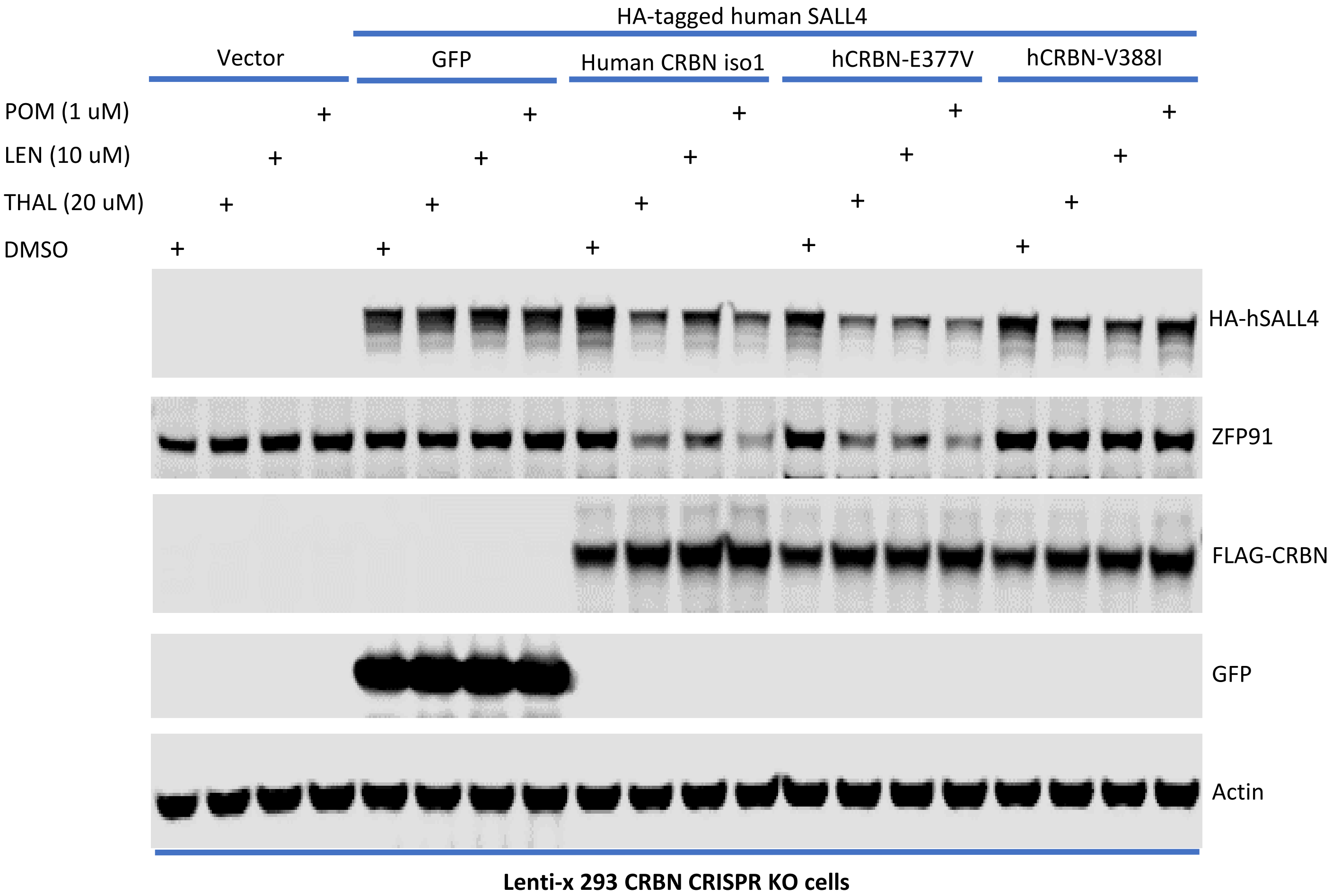

Supplementary Figure 12. Human CRBN E377V mutation abolished thalidomide-, lenalidomide-, and pomalidomide-induced degradation of human SALL4. enti-x-293 CRBN-/- cells stably expressing human SALL4 were transiently transfected with vectors expressing GFP or human CRBN variants and treated with 20 µM thalidomide, 10 µM lenalidomide, 1 µM pomalidomide, or 0.1% DMSO for 24 hours. Lenti-x-293 CRBN-/- cells transfected with empty vector were used as a negative control. The cells were then lysed and characterized by immunoblotting analysis for SALL4, ZFP91, GFP, CRBN, and β-actin. **Method:** Lenti-x 293 CRBN CRISPR KO (CRBN-/-) cells were maintained in Dulbecco's Modified Eagle's medium (DMEM; Invitrogen) supplemented with 10% fetal bovine serum (FBS; Invitrogen). All cell lines were confirmed mycoplasma-negative using the MycoAlert Mycoplasma Detection Kit (Lonza). One million of lenti-x-293 CRBN-/- cells stably expressing human SALL4 was transiently transfected with 4 µg of pcDNA3-GFP, pcDNA3-FLAG-CRBN WT (isoform 1), pcDNA3-FLAG-CRBN E377V (isoform 1) or pcDNA3-FLAG-CRBN V388I (isoform 1) in 6-well plates. Twenty-four after transfection, cells were treated with DMSO, thalidomide, lenalidomide or pomalidomide at the indicated concentrations for 24 hours. Cells were then harvested and lysed, and whole cell extract was subjected to immunoblotting analysis. Cells were washed with ice-cold 1X PBS and lysed in 50mM Tris-HCl [pH 7.6], 150 mM NaCl, 1% Triton X-100, and 1x Complete ULTRA protease inhibitor cocktail and PhosSTOP phosphatase inhibitor cocktail. Whole cell extracts were collected after centrifugation at top speed for 10 minutes, resolved by sodium dodecyl sulfate polyacrylamide gel electrophoresis, transferred onto a nitrocellulose membrane using the Turboblot system (Bio-Rad), and probed with the indicated primary antibodies. Bound antibodies were detected with IRDye®-680 or -800 conjugated secondary antibodies using a LI COR scanner. Primary antibodies: Mouse anti-HA monoclonal antibody (Biolegend, Cat # 901503); Rabbit anti-human CRBN monoclonal antibody (Celgene, Cat # CRBN65) ; Mouse anti-GFP monoclonal antibody (Santa Cruz, Cat #SC-9996); Rabbit anti-ZFP91 polyclonal antibody (LS Bio, Cat#LS-B14788); Mouse anti-human Actin monoclonal antibody (Sigma, Cat # A5316). Uncropped gels can be found in Supplementary Fig. 13.

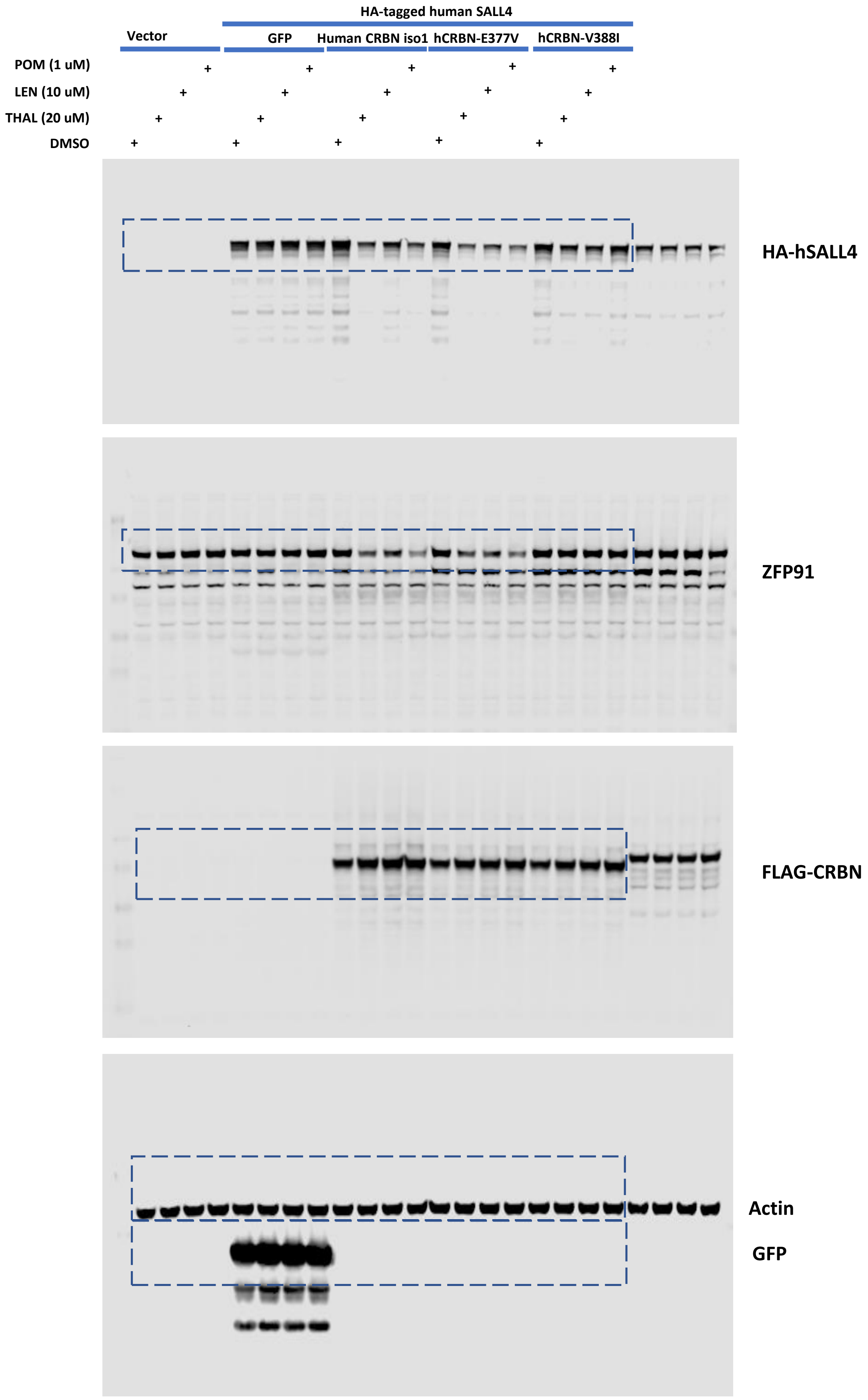

Supplementary Figure 13. Uncropped Western blot gels.
